# Chromosome conformation signatures define predictive markers of inadequate response to methotrexate in early rheumatoid arthritis

**DOI:** 10.1186/s12967-018-1387-9

**Published:** 2018-01-29

**Authors:** Claudio Carini, Ewan Hunter, Scottish Early Rheumatoid Arthritis Inception cohort Investigators, Duncan Porter, Iain McInnes, David Reid, Stuart H. Ralston, Neil Basu, Leon Collis, Carl S. Goodyear, Janet Liversidge, Caron Paterson, Jane Hair, Sharon Kean, Ashley Gilmour, Margaret Duncan, Susan Fraser, Lisa Hutton, John Harvie, Vinod Kumar, Mike McMahon, Robin Munro, John Larkin, Neil McKay, John McLaren, David M. Reid, Duncan Porter, Ruth Richmond, Gillian Roberts, Sarah Saunders, Hilary Wilson, Aroul S. Ramadass, Jayne Green, Alexandre Akoulitchev, Iain B. McInnes, Carl S. Goodyear

**Affiliations:** 1 Pfizer Inc., Cambridge, USA; 2 Oxford BioDynamics Plc, Oxford, UK; 3 Institute of Infection, Immunity and Inflammation, University of Glasgow, Glasgow, UK; 4 Department of Asthma, Allergy & Lung Biology, GSTT Campus, King’s College School of Medicine, London, UK

**Keywords:** Early rheumatoid arthritis, Methotrexate, Rheumatoid arthritis, DMARDs (synthetic), Precision medicine drug response biomarkers, Methotrexate (MTX), Chromatin conformation signatures (CCS), Expression quantitative trait loci (eQTL)

## Abstract

**Background:**

There is a pressing need in rheumatoid arthritis (RA) to identify patients who will not respond to first-line disease-modifying anti-rheumatic drugs (DMARD). We explored whether differences in genomic architecture represented by a chromosome conformation signature (CCS) in blood taken from early RA patients before methotrexate (MTX) treatment could assist in identifying non-response to DMARD and, whether there is an association between such a signature and RA specific expression quantitative trait loci (eQTL).

**Methods:**

We looked for the presence of a CCS in blood from early RA patients commencing MTX using chromosome conformation capture by EpiSwitch™. Using blood samples from MTX responders, non-responders and healthy controls, a custom designed biomarker discovery array was refined to a 5-marker CCS that could discriminate between responders and non-responders to MTX. We cross-validated the predictive power of the CCS by generating 150 randomized groups of 59 early RA patients (30 responders and 29 non-responders) before MTX treatment. The CCS was validated using a blinded, independent cohort of 19 early RA patients (9 responders and 10 non-responders). Last, the loci of the CCS markers were mapped to RA-specific eQTL.

**Results:**

We identified a 5-marker CCS that could identify, at baseline, responders and non-responders to MTX. The CCS consisted of binary chromosome conformations in the genomic regions of IFNAR1, IL-21R, IL-23, CXCL13 and IL-17A. When tested on a cohort of 59 RA patients, the CCS provided a negative predictive value of 90.0% for MTX response. When tested on a blinded independent validation cohort of 19 early RA patients, the signature demonstrated a true negative response rate of 86 and a 90% sensitivity for detection of non-responders to MTX. Only conformations in responders mapped to RA-specific eQTL.

**Conclusions:**

Here we demonstrate that detection of a CCS in blood in early RA is able to predict inadequate response to MTX with a high degree of accuracy. Our results provide a proof of principle that a priori stratification of response to MTX is possible, offering a mechanism to provide alternative treatments for non-responders to MTX earlier in the course of the disease.

**Electronic supplementary material:**

The online version of this article (10.1186/s12967-018-1387-9) contains supplementary material, which is available to authorized users.

## Background

Rheumatoid arthritis (RA) is an autoimmune inflammatory disorder driven by interacting genetic, epigenetic and environmental factors [1–3]. The diagnosis of RA prompts early initiation of methotrexate (MTX), the first choice of disease modifying anti-rheumatic drug (DMARD) as recommended by European League against Rheumatism (EULAR) and American College of Rheumatology (ACR) ‘treat-to-target’ strategy (target being remission or low disease activity). This approach has substantially improved outcomes in the last decade [4–6]. However, approximately 35–59% of patients do not achieve clinically meaningful responses after starting MTX [7]. Predicting response to MTX has been one of the main challenges in RA management for over two decades [8]. Delay of effective treatment has clinical implications as response to MTX is the most significant predictor of long-term outcome in RA [9–11]. The ability to identify biomarkers able to predict inadequate response has thus far proven challenging [7, 12–15]. Several attempts have been made to develop diagnostics able to predict MTX-response [16–23] (Additional file 1: Table S1) however, most studies have failed.

RA is dependent on the interaction of genetic and environmental factors [24, 25]. Epigenetic markers are closely linked to transcriptional regulation and may reflect pathogenic changes associated with disease states [26–30]. The first evidence suggesting that epigenetic mechanisms may play a role in autoimmune diseases came from studies performed by Richardson et al. looking at the effect of DNA methyltransferase inhibitor 5-azacytidine [31]. Other studies have also reported aberrant methylation in RA [32–38]. Interestingly, MTX treatment also plays a role in epigenetic regulation in RA [39–41]. Gene expression in mammals is regulated by non-coding elements that can impact physiology and disease; the principle mechanism of regulation is through the architectural status at both coding and non-coding genomic regions [26]. Under external perturbations, genomic regions can alter their 3-dimensional structure as a way of functional regulation of gene expression [42]. These structural changes can be measured by EpiSwitch, a high-throughput molecular technique that analyzes the spatial organization of genomic loci in a cell [43–45]. As multiple genomic regions contribute to phenotypic differences through changes in genomic architecture [26], this approach allows for the development of a chromosomal conformation signature (CCS) of alterations in genomic architecture between two states (disease vs. non-disease, pre-treatment vs. post-treatment). The evaluation of long range chromosome interactions has provided useful blood-based biomarkers in oncology and other non-rheumatic diseases [44–48]. CCS offer a stable, binary readout of cellular states and represent an emerging class of biomarkers [49]. Here, we used a chromosomal architecture based approach to predict the response to MTX by developing a blood-based classifier based on a CCS. We hypothesized that interrogation of genomic architectural changes in early RA patients would define a functional endotype able to guide clinical decision making.

## Methods

### Patient population

The SERA study is a national prospective inception cohort of patients with new RA or undifferentiated arthritis, together with age-gender matched controls [50, 51]. We used 67 SERA patients for the discovery/validation phases (plus 34 healthy controls) and an additional 19 patients from an independent cohort, who were enrolled in a blinded validation study (demographic and clinical characteristics are shown in Additional file 1: Tables S2–S4). Participants fulfilled the 2010 ACR/EULAR RA criteria at recruitment and commenced monotherapy with MTX. Importantly, patients were treated to standard of care according to SIGN Guidelines (http://www.sign.ac.uk/pdf/sign123.pdf) reflecting the true to life, population wide cohort design of SERA. Patients were treated with standard therapy, which included intra-articular or intra-muscular glucocorticoids, offered as required in the absence of low dose oral glucocorticoids. Clinical data to calculate a range of composite clinical outcomes were obtained at baseline and after 6 months of MTX treatment (Additional file 1: Tables S2–S4). DAS (Disease Activity Score) response was calculated on the basis of either DAS28 (ESR) or DAS28 (CRP) change to meet the following criteria for response. Responders (R) were defined as those achieving DAS28 remission (DAS28 < 2.6) or a good response (DAS28 improvement of > 1.2 and DAS28 ≤ 3.2) at 6 months. Non-responders (NR) were defined as patients who had no improvement in DAS28 (≤ 0.6) by 6 months. For consistency, we then calculated a CDAI (Clinical Disease Activity Index) response for each patient and used this outcome measure to ensure comparability of clinical response across all patients in the analysis [52, 53]. Baseline peripheral blood samples with EDTA were collected and centrifuged to generate a buffy layer, stored at − 80 °C. Local ethics committees approved the SERA protocol and all participants gave informed consent before enrollment.

### Identification of markers by EpiSwitch™ and probe design

A pattern recognition algorithm was used to annotate the human genome for sites with the potential to form long-range chromosome conformations. The proprietary EpiSwitch pattern recognition software, based on Bayesian-modeling, provides a probabilistic score that a region is involved in long-range chromatin interactions [44, 45]. Sequences from 123 gene loci (Additional file 1: Table S5), selected based on a systematic literature review for genes that have been associated with RA [35, 54–57], were processed through the pattern recognition software to generate a list of the 13,322 chromosomal interactions to be screened for association with response to MTX in RA. Sixty 60-mer oligonucleotide probes were designed to interrogate these potential interactions and uploaded as a custom array to the Agilent SureDesign website. Importantly, each probe was present in quadruplicate on the EpiSwitch™ microarray. To subsequently evaluate a potential CCS, nested PCR (EpiSwitch™ PCR) was performed using sequence-specific oligonucleotides designed using Primer3. Oligonucleotides were tested for specificity using oligonucleotide specific BLAST.

### Preparation of genomic templates

Chromatin with intact chromosome conformations from 50 µl of each blood sample was extracted using the EpiSwitch assay following the manufacturer’s instructions (Oxford BioDynamics Ltd.) [43, 45, 46]. The methods used to perform an EpiSwitch microarray and EpiSwitch PCR detection are described in detail in Supplementary Methods.

### Statistical analysis

GraphPad Prism and SPSS were used for all statistical analyses of clinical data. The R statistical environment was used to analyze the CCS data. The Fisher’s exact test (for categorical variables), the t test for independent samples (for continuous normally distributed variables), and the Mann–Whitney U test (for continuous variables without normal distribution) were used to identify differences. The level of statistical significance was set at p ≥ 0.05, and all tests were 2-sided. A full description of the statistical methods used to identify the CCS can be found in Additional file 1: Additional Methods.

### Mapping of CCS locations to expressed quantitative trait locus (eQTL)

The genomic locations of the five markers (IFNAR1, IL-21R, IL-23, CXCL13 and IL-17A) in the CSS were compared with the Walsh et al. [58] eQTLs data using a Window function within the Bedtools suit of genomic analysis functions [59]. Only eQTLs that were within a genomic window of 100 bp to 2 Kbp were considered when mapping to the CCS locations.

## Results

### Patient characteristics

**Fig. 1:**
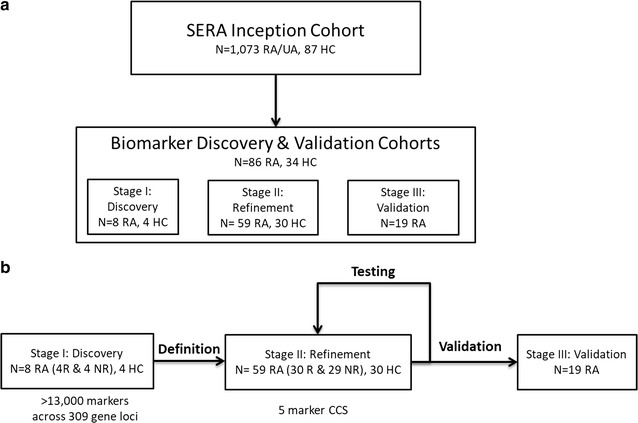
Study design. **a** Samples used for biomarker discovery and validation. A subset of patient samples from the SERA Inception Cohort (86 RA patients and 34 HC) were used to discover and validate the MTX response biomarker. **b** Workflow for discovery and validation of the epigenetic stratifying biomarker. For Stage I and Stage II biomarker discovery and testing, clinical samples from MTX-treatment naïve patients were provided after confirmation of response by SDAI. In Stage I, an initial panel of 13,322 potential biomarkers was refined to a 5-marker chromosomal conformation signature (CCS). In Stage II, the disease specific nature of the biomarker panel was confirmed by stratification against HC and further testing was performed on 59 RA patients (29 R and 30 NR) and 30 HC. Final validation of the biomarker panel was done on an independent, blinded cohort of 19 RA patients

Demographic and clinical characteristics was captured for the early RA patient groups (Stages I, II and III) at the time of recruitment and at 6 months following MTX therapy (Additional file 1: Table S2–S4). A total of 86 early RA patients and 34 healthy controls (HC) were included. 67 were used to develop the CCS, and an independent, blinded cohort of 19 patients used in validation (Fig. 1). The biomarker discovery pipeline comprised 3 stages (Fig. 1). Stage I, the discovery phase was performed using 8 early RA patients (4 MTX-NR and 4 MTX-R) and 4 HC. Stage II, conducted to test the CCS involved 59 early RA patients (30 MTX-NR and 29 MTX-R) and 30 HC. Stage III validation used an independent, blinded cohort of 19 early RA patients (Fig. 1).

### Initial definition of the CCS in early RA

**Fig. 2:**
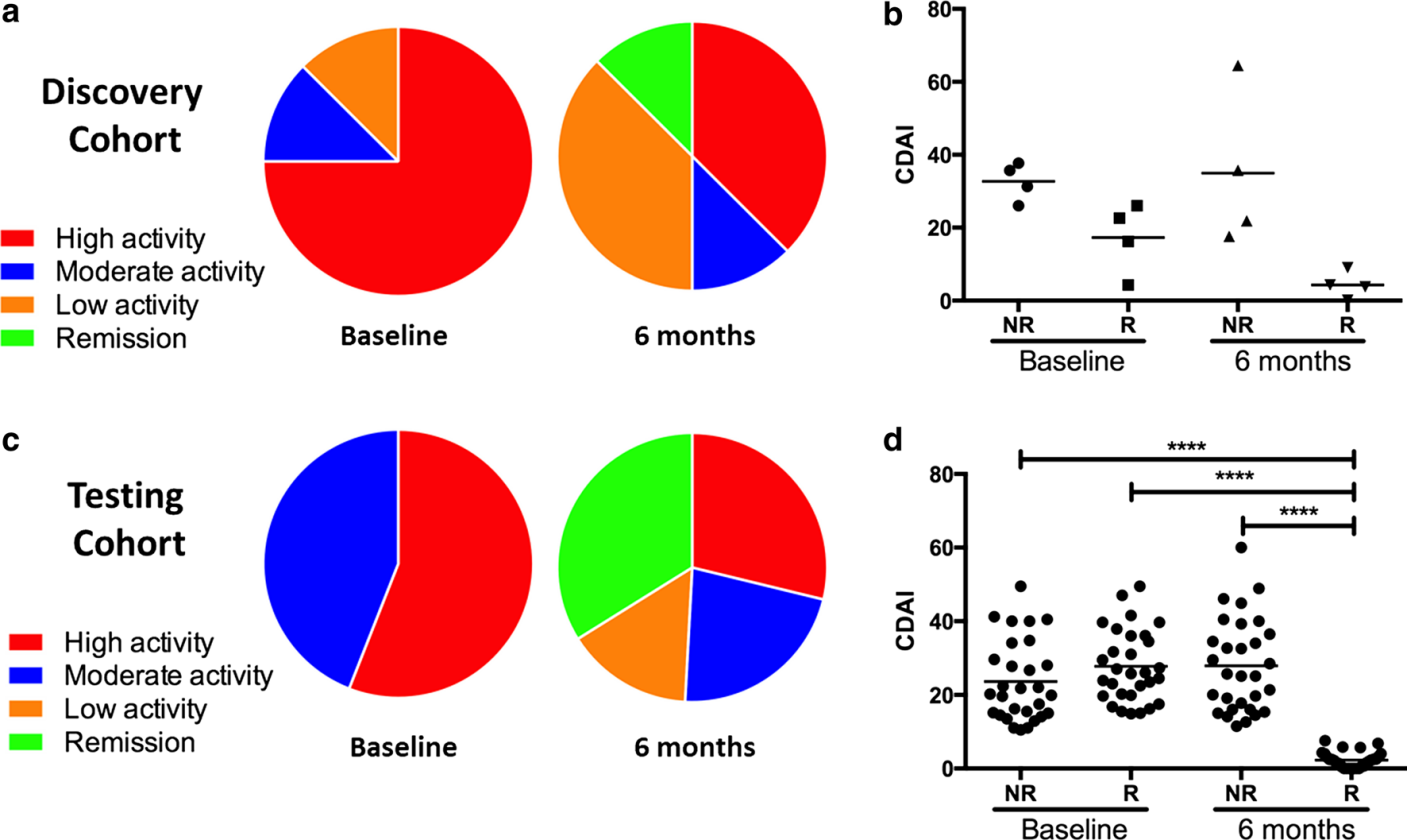
Clinical Data for Discovery and Testing Cohorts. Discovery cohort: (**a**, **b**). **a** Breakdown of disease severity by CDAI scores for the discovery cohort at Baseline and 6 months. **b** Change in CDAI scores between responders (R) and non-responders (NR) at Baseline and 6 months following MTX therapy. Testing cohort: (**c**, **d**). **c** Breakdown of disease severity by CDAI scores for the testing cohort at Baseline and 6 months. **d** Change in CDAI scores between responders (R) and non-responders (NR) at Baseline and 6 months following MTX therapy. Significant reductions in CDAI scores were seen between R and NR at Baseline compared to R at 6 months as well as between NR and R at 6 months (****p<0.01). For CDAI scores see Additional file 1: Tables S2, S3

To identify an initial set of common epigenetic profiles in early RA patients, 123 genetic loci (Additional file 1: Table S5) previously associated with RA [35, 54–57] were selected and annotated with chromosome conformation interactions predicted using the EpiSwitch in silico annotation package [44–46]. The in silico prediction generated 13,322 high-confidence chromosome interaction candidates, with an average of 99 per loci (99 ± 64; mean ± SD) (Additional file 1: Table S5). These candidates were used to generate a bespoke discovery CCS array (Additional file 1: Table S5) to screen baseline whole blood samples from 4 MTX-R and 4 MTX-NR (Fig. 1; Additional file 1: Table S2), and 4 HC. Age, gender makeup, DAS28, baseline CDAI and MTX doses were similar in R and NR patients (Fig. 2a, b; Additional file 1: Table S2). We identified 922 statistically significant chromosomal interactions that differentiated R, NR and HC. Of the 922 lead interactions, 420 were associated with NR, 210 with R and 159 with HC. A stepwise hierarchical clustering approach reduced the number of significant interactions from 922 to a 30-marker profile that effectively stratified patients (Additional file 1: Figure S1, Table S6).

### Refinement of the CCS

The 30 interactions identified in the initial screen were narrowed to a smaller pool using a second SERA-derived early RA patient cohort of 59 patients (30 MTX-R and 29 MTX-NR) and 30 HCs (Fig. 2c, d; Additional file 1: Table S3). Employing a stepwise approach using the EpiSwitch PCR platform (Additional file 1: Figure S1, blood samples from 24 RA patients (12 R and 12 NR) were analyzed for the interactions that best differentiated between R and NR. This resulted in a set of 10 best discriminatory interactions (Additional file 1: Table S6). These ten interactions were used to probe blood samples from an additional set of 35 RA (18 R and 17 NR). Using logistical regression on the combined data from all patients used to refine the CCS (that is 30 R and 29 NR), we identified five conformations within the IFNAR1, IL-21R, IL-23, IL-17A, and CXCL13 loci which provided the final CCS for response to MTX (Additional file 1: Figures S2–S6, Table S8). The regression coefficients and odds ratio of the logistic regression model are shown in Table 1.

**Table 1:** Coefficients of the logistic regression model for predicting efficacy of MTX monotherapy in baseline samples based on retrospective annotation at 6 months

Locus	Regression coefficient	Odds ratio (95% CI)
IFNAR1	2.0	7.6 (1.4–57)
IL-17A	− 3.2	0.04 (0.001–0.41)
CXCL13	− 1.9	0.15 (0.02–0.69)
IL-21R	2.2	9.0 (2.1–49)
IL-23	1.4	4.2 (0.78–33)

The model provided a prediction probability score for R and NR, ranging from 0.0098 to 0.99 (0 = responder, 1 = non-responder). The cut-off values were set at ≤ 0.30 for R and ≥ 0.70 for NR. The score of ≤ 0.30 had a true positive rate (sensitivity) of 89% (95% confidence interval [95% CI] 72–98%), while a score of ≥ 0.70 had a true negative response rate (specificity) of 87% (95% CI 70–96%). The number of observed and predicted patients per response category is shown in Table 2. With the CCS classifier, 53 patients (90%) were correctly classified as either R or NR.

**Table 2:** Observed and predicted number of R and NR to MTX monotherapy at 6 months using the CCS classifier

Observed response	Predicted response		
	Non-responder	Undefined	Responder
Non-responder	25	1	3
Responder	3	7	20

Cut off levels were chosen based on the logistic model probabilities of response to MTX of (approximately) > 0.70 for NR and < 0.3 for R. NR and R were defined as described in Additional file 1: Additional Methods

### Testing the performance of the CCS

**Fig. 3:**
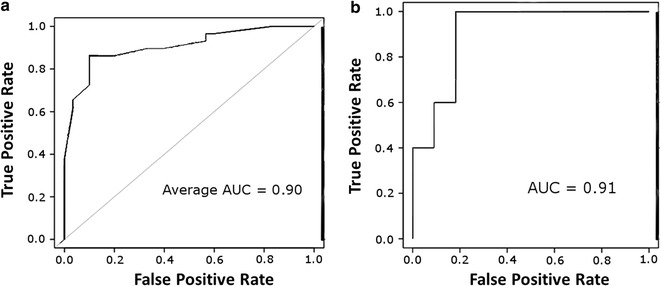
CCS performance on randomized subsets of testing cohort and validation cohort. **a** Receiver operating characteristic (ROC) plots for 150 runs of the EpiSwitch™ logistic classifier. Data for the 59 patient cohort was randomised 150 times using the WEKA sample randomisation function. This reorders the data prior to splitting in developing the training set, ensuring that the same starting point for the classifier is not used and allowing multiple accuracy calculations for the same data. The average area under the curve (AUC) for the 150 classifier runs was 0.90. The plot is the average ROC from the 150 test results. **b** Receiver operating characteristic (ROC) plots for the EpiSwitch™ logistic classifier run on the blinded validation cohort of 19 RA patients. The classifier had a sensitivity of 75.0% and specificity of 85.7% with an AUC of 0.91

To test the accuracy and performance robustness of the CCS classifier to discriminate non-responders to MTX in early RA prior to treatment, data from the 59 early RA patient cohort used to develop the classifier was split into 150 different training and test sets by random data re-sampling using fivefold cross-validation. While this analysis was not intended as a true validation since the classifier was tested on the same cohort used to develop it, this provided an interim measure of the classifiers ability to discriminate R and NR. The average area under the curve (AUC) of the model was 90.6% (95% CI 87–100%), with an average sensitivity for NR of 89.7% and an average specificity for R of 90.0% (Fig. 3a). To determine the predictive capability of the CCS classifier, the average model accuracy statistics were adjusted for population R/NR to MTX [60]. Using a 55% MTX response rate, the positive predictive value (PPV) was 88% while the negative predictive value (NPV) was 86.4%. When the response rate was adjusted to 60%, this decreased the PPV to 85.7% while increasing the NPV to 88.6%.

### Independent blinded validation

To validate the performance of the CCS, a blinded analysis was undertaken on an additional 19 early RA patients from an independent cohort selected from SERA (9 R and 10 NR) (Additional file 1: Table S4, Figure S8). This validation cohort had similar clinical characteristics to those used to develop the CCS with respect to age, gender, RA severity (as measured by CDAI, DAS28-CRP, and DAS28-ESR) and MTX dosage (Additional file 1: Tables S3, S4). The analysis of the validation cohort was done using a blinded analysis, where the actual response calls were not known until after the identification of R and NR using the CCS classifier. In this independent cohort, we obtained a PPV of 90% (95% CI 59–98%), and a NPV rate of 66.7% (95% CI 42–85%), sensitivity of 75.0% (95% CI 43–95%) and specificity of 85.7% (95% CI 42–97%). The area under the receiver operating characteristic curve (AUROC) was 0.91 (Fig. 3b). The number of observed and predicted patients per response category is shown in Table 3. The strong NPV statistics for the blinded samples confirms the accuracy and robust stratification power of the CCS for non-response to MTX prior to treatment.

**Table 3:** CCS classifier performance for predicting non-response to MTX in a blinded cohort of early RA patients

Observed response	Predicted response	
	Non-responder	Responder
Non-responder	9	1
Responder	3	6

### Biological and functional characterization of the genomic loci making up the CCS

**Fig. 4:**
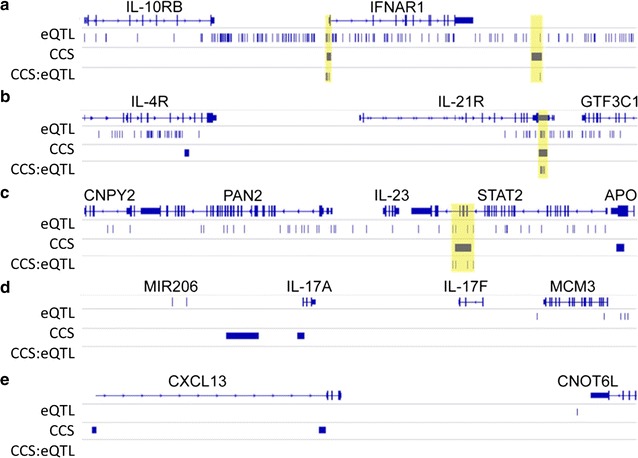
Chromosome conformations in MTX responders map to RA eQTLs. Bedtools shot of CCS markers mapped within 200 bp of previously identified RA eQTLs. The overlapping CCS:eQTLs are highlighted in yellow. **a** The CCS regions associated with the IFNAR1 locus on chromosome 21 map to 6 eQTLs. **b** The CCS regions associated with the IL-21R locus on chromosome 16 map to 21 eQTLs. **c** The CCS regions associated with the IL-23 locus on chromosome 12 map to 4 eQTLs. **d** The CCS regions associated with the IL-17A locus on chromosome 6 do not map to any eQTLs. **e** The CCS regions associated with the CXCL13 locus on chromosome 4 do not map to any eQTLs

The five genomic loci (IFNAR1, IL-21R, IL-23, IL-17A, and CXCL13) that make up the CCS fall within genes that encode for proteins involved in the immune response. Recently, eQTLs that overlap with enhancer elements have been identified in RA patients with moderate to severe disease and inadequate response to MTX [58]. eQTLs are genomic loci that contribute to variation in expression levels of mRNAs. We mapped the genomic locations of the five loci in the CCS to these previously published eQTLs [58]. The two genomic sites that corresponded to the junction of each CCS locus were mapped to eQTLs that were within 100, 200, 500, 1000 or 2000 base pairs (Additional file 1: Table S9). There was enrichment of eQTLs co-localized with the R-associated CCS loci (IFNAR1, IL-21R and IL-23). In contrast, no co-localization was observed in the NR-associated CCS loci (IL-17A and CXCL13) (Fig. 4).

## Discussion

To address an unmet clinical need of predicting those RA patients who will not respond to MTX, we analyzed the CCS in whole blood taken at baseline from SERA-RA patients using a well-established method of analyzing long-range chromatin interactions [43–45]. We identified a 5-marker panel consisting of chromosomal conformations in the genomic loci of IFNAR1, IL-21R, IL-23, IL-17A, and CXCL13 that could identify R and NR to MTX with 90% sensitivity in an independent blinded validation cohort. This could facilitate earlier access to more effective therapies, thus avoiding disease progression, unnecessary exposure to potentially toxic drugs and diminished quality of life.

Historically, identifying predictive biomarkers for MTX response has been difficult [7, 12–15]. While clinical predictors of RA disease are well established [61, 62], they do not correlate well with response to treatment at the individual level [13]. 35–59% of patients do not achieve clinically meaningful response after starting MTX [7, 63]. In the SERA study, only 30% of patients responded to MTX monotherapy [50]. While the results presented here provide a proof-of-concept and further validation is warranted, the EpiSwitch technology has several attractive features from the standpoint of a biomarker that can be used clinically [49]. First, it requires a very small amount of blood sample (typically 50 μl). Second, it utilizes an established laboratory methodology and readouts (qPCR). Last, the turnaround time is short (~ 8 h). Thus, once further validated, the approach described here faces low barriers to clinical adoption. The CCS defined a signature that suggests epigenomic control over loci with a central role in the IL-17/IL-23 axis, with the two most informative long-range chromatin interactions for predicting MTX-NR coming from IL-17A and CXCL13 loci. In agreement with our finding, earlier reports have suggested that IL-23, IFNAR1 and IL-21R are predictors of positive response in other diseases [64–66], while IL-17A and CXL13 are predictors of poor outcome and increased disease severity [67–71]. Several studies support the notion that IL-23/Th17 axis drive inflammation in chronic diseases and perhaps plays a role in the response to immunomodulating drugs [72–75]. Interestingly, no loci previously associated with MTX metabolism were implicated in our study, indicating that the response to MTX was independent of how the drug is known to be metabolized [20, 76].

Our results are consistent with previous data reporting the presence of regulatory elements (i.e. eQTL) only in the context of inflammatory diseases and not in healthy controls [77].

Recently, Walsh et al. reported that eQTL mapped from RA are particularly enriched in enhancer regions of disease related cell types such as T and B cells [58]. Previous studies have recognized that the eQTL are highly specific to different leucocyte subsets [78]. To explore the concordance of CCS data with existing genetic regulatory datasets, we mapped the genomic locations of the regions in the CCS against reported RA-specific eQTL [58]. This revealed a high level of co-localization of R-specific loci (IFNR1, IL-21R, and IL-23) to RA-specific eQTL. Notably, this level of concordance was not observed in NR loci (IL-17 and CXCL-13). The increased association of RA eQTLs with CCS regions observed in R, but not in NR, suggest dysregulation at the level of regulatory 3D genome architecture. It has been reported that eQTL are associated with expression of mRNA transcripts, with concomitant effects on protein levels [79–82]. This leaves open the possibility that the association of eQTL with CCS observed in RA-R result from phenotypic consequences due to the effects of mRNA and protein expression levels. However, the impact of eQTL on protein levels remains poorly understood.

It is now clear that the developing immune response is influenced by genetic and epigenetic factors [41]. IFNAR1, IL-21R and IL-23 loci have been reported to play a key role in the pathogenesis of RA [83–85] and Th17 cells are implicated in pathogenesis especially in the pre-RA phase [68, 72, 73]. Whether the CCS differences we observed represent changes impacting the pre-RA phase or are acquired during the early phase of RA is at present unclear. We hypothesize that the different genomic architecture observed for R and NR might reflect differences in epigenetic host responses to early pathogenetic events, or particular environmental exposures. We anticipate that the association observed between CCS and eQTL in RA patients may be important to understand the heterogeneity of the response observed between individuals. Furthermore, eQTLs present in active inflammatory diseases can disappear after treatment [77]. It would therefore be useful to determine whether the concordance of the CCS and eQTL observed in our study in treatment naïve RA responders will be linked only to the disease or change after MTX treatment. Moreover, it would be interesting to see whether the reported eQTL are linked to the disease state and/or the level of inflammation [68]. Our data indicate that the mapping of the QTL can reveal an altered biological status in R and NR, however further studies are needed to confirm this.

The samples used in this study came from the SERA study, a large, multi-institutional investigational program designed to identify predictive markers of RA [50]. Patients enrolled in the SERA study were carefully characterized for clinical phenotype (the majority of the patients were Caucasians, non-smokers with seropositive established disease), longitudinal follow-up of outcomes and blood samples were stored following a Standard Operating Procedure. This combination of clinical rigor and quality assurance of the inputs to the CCS are particular strengths of this study. An additional strength of the study is the approach that was used to generate the CCS. We focused on the discovery and establishment of a molecular signature using an approach informed by current biological knowledge. We evaluated a network of loci that had plausible pathophysiological relevance in RA via synovial pathogenesis studies, GWAS association and postulated MTX pharmacogenetics [35, 54–57, 86]. The step-wise selection of biomarkers used in this study, coupled with the strict separation of discovery and validation cohorts, was performed to prevent marker and model over-fitting. The robust statistical properties of the CCS classifier are another advantage of the approach presented here. Using epigenetic markers, which provide a binary readout (presence or absence) and are stable in isolated whole blood, provides high efficiency stratification. Statistical power analysis confirmed that the sample size was adequate for the development and evaluation of the signature, a critical step in biomarker development and aligned with successful development of companion diagnostics in limited size cohorts in other indications [87]. A further strength to our findings and applicability in the clinical setting is the ability to identify this molecular signature in the whole blood by using a drop of blood.

Some of the caveats associated with our findings are the relatively modest sample size and the cellular heterogeneity present in the whole blood. While the sample size for the validation cohort was not as large as previous studies seeking to identify a biomarker for MTX non-response (Additional file 1: Table S1), the use of chromosome conformations as a readout, which can generate robust signatures in smaller cohorts, provide confidence in the approach [88, 89]. The heterogeneity of cell populations is an issue that has implications for any analysis in whole blood. In RA, it is known that there is significant heterogeneity in cell populations present in the blood of RA patients [90]. While outside the scope of the current study, future studies that look at the CCS in distinct cell types in whole blood may shed greater light on the similarities and/or differences exhibited within individual populations. A final caveat of our study was limited in that it could not determine whether the observed epigenetic marks are causal or consequential (secondary to the inflammatory response). Future studies looking at larger patient sets as well as the inclusion of individuals treated with other DMARDs, including biologics, are warranted.

## Conclusions

The ability to identify individuals who are unlikely to respond to a specific therapeutic agent has the potential to not only reduce side-effects to treatments that will provide no benefit but also accelerate their journey through the treatment algorithm and thus increase likelihood of a positive treatment outcome. Given the high worldwide prevalence of RA, a stratifying signature for MTX response offers direct and practical benefits. The validated classifier described here, which is based on five conditional binary biomarkers detected in the blood, offers cost-effective detection by an established ISO-certified industrial platform and the practicality that this method to stratify patients has the potential to be routinely available within clinical practice. This could be a first step toward personalized medicine in RA.

## Additional file

**Additional file 1: Note.** Scottish Early Rheumatoid Arthritis inception cohort (SERA). **Table S1.** Previous studies on prediction of MTX response in RA. **Table S2.** Patient characteristics—discovery cohort. **Table S3.** Patient characteristics—test cohort. **Table S4.** Patient characteristics—blinded validation cohort. **Additional Methods.** Statistical analysis and details of assays used to generate the CCS. **Table S5.** Selected genes for EpiSwitch Array. **Figure S1.** Design for discovery and validation of epigenetic stratifying biomarker signature for SERA patients. **Table S6.** Stepwise marker selection. **Table S7.** 65 Selected genes from EpiSwitch Array analysis. **Table S8.** 12 Selected genes from EpiSwitch PCR. **Figure S2.** Graphical representation of the genomic co-ordinates of the CXCL13 CCS marker associated with non-response to MTX. **Figure S3.** Graphical representation of the genomic co-ordinates of the IL- 17A CCS marker associated with non-response to MTX. **Figure S4.** Graphical representation of the genomic co-ordinates of the IFNAR1 CCS marker associated with non-response to MTX. **Figure S5.** Graphical representation of the genomic co-ordinates of the IL- 21R CCS marker associated with non-response to MTX. **Figure S6.** Graphical representation of the genomic co-ordinates of the IL-23 CCS marker associated with non-response to MTX. **Figure S7.** Clinical characteristics of independent blinded validation cohort. **Table S9.** CCS markers associated with eQTLs.

## Abbreviations

GWAS: genome wide association study
EULAR: European League Against Rheumatism
DAS: Disease Activity Index
ESR: erythrocyte sedimentation rate
CRP: C reactive protein
CDAI: Clinical Disease Activity Index
R: responders
NR: non-responders
HC: healthy controls
RA: rheumatoid arthritis
MTX: methotrexate
CCS: chromatin conformation signatures
SERA: Scottish Early Rheumatoid Arthritis
TNR: true negative response
DMARDS: disease modifying anti-rheumatic drug
ACR: American College of Rheumathologists
eQTL: expression quantitative trait loci
