# Supplementary material for: Chromosome conformation signatures define predictive markers of inadequate response to methotrexate in early rheumatoid arthritis
Source: J Transl Med. 2018 Jan 29;16:18. doi: 10.1186/s12967-018-1387-9 (PMC5789697; doi:10.1186/s12967-018-1387-9)
Supplement: Supplementary file 1 — Additional file 1: Note. Scottish Early Rheumatoid Arthritis inception cohort (SERA). Table S1. Previous studies on prediction of MTX response in RA. Table S2. Patient characteristics—discovery cohort. Table S3. Patient characteristics—test cohort. Table S4. Patient characteristics—blinded validation cohort. Additional Methods. Statistical analysis and details of assays used to generate the CCS. Table S5. Selected genes for EpiSwitch Array. Figure S1. Design for discovery and validation of epigenetic stratifying biomarker signature for SERA patients. Table S6. Stepwise marker selection. Table S7. 65 Selected genes from EpiSwitch Array analysis. Table S8. 12 Selected genes from EpiSwitch PCR. Figure S2. Graphical representation of the genomic co-ordinates of the CXCL13 CCS marker associated with non-response to MTX. Figure S3. Graphical representation of the genomic co-ordinates of the IL- 17A CCS marker associated with non-response to MTX. Figure S4. Graphical representation of the genomic co-ordinates of the IFNAR1 CCS marker associated with non-response to MTX. Figure S5. Graphical representation of the genomic co-ordinates of the IL- 21R CCS marker associated with non-response to MTX. Figure S6. Graphical representation of the genomic co-ordinates of the IL-23 CCS marker associated with non-response to MTX. Figure S7. Clinical characteristics of independent blinded validation cohort. Table S9. CCS markers associated with eQTLs. [file 12967_2018_1387_MOESM1_ESM.docx]

**Additional Materials:**

**Additional Note: Scottish Early Rheumatoid Arthritis Inception cohort (SERA)**

Steering Committee Members: Duncan Porter^1^ and Iain McInnes^1^ (Chief Investigators); David Reid^2^, Stuart H. Ralston^3^, Neil Basu^2^, Leon Collis^4^, Carl S. Goodyear^1^, Janet Liversidge^2^

Study Team: Caron Paterson^1^, Jane Hair^5^, Sharon Kean^6^, Ashley Gilmour^1^

Investigators:

Margaret Duncan, Ayr Hospital

Susan Fraser, Southern General Hospital, Glasgow Lisa Hutton, Inverclyde Royal Hospital

John Harvie, Raigmore Hospital, Inverness

Vinod Kumar, Ninewells Hospital, Dundee

Mike McMahon, Dumfries & Galloway Royal Infirmary Robin Munro, Wishaw General Hospital

John Larkin, Victoria Infirmary Glasgow

Neil McKay, Western General Hospital, Edinburgh John McLaren, Whyteman's Brae Hospital, Fife David M Reid, Aberdeen Royal Infirmary

Duncan Porter, Gartnavel General Hospital, Glasgow

Ruth Richmond, Borders General Hospital, Melrose Gillian Roberts, Vale of Leven Hospital

Sarah Saunders, Glasgow Royal Infirmary

Hilary Wilson, Stobhill Hospital, Glasgow

1. University of Glasgow, Institute of Infection, Immunity and Inflammation, Glasgow UK

1. School of Medicine and Dentistry, University of Aberdeen, Aberdeen, UK
2. Rheumatology and Bone Diseases Unit, Centre for Genomic and Experimental Medicine, MRC Institute of Genetics and Molecular Medicine, Western General Hospital, University of Edinburgh, UK
3. Pfizer Inc. Cambridge, USA
4. NHS Greater Glasgow & Clyde Bio-repository, Pathology Department, Southern General Hospital, Glasgow, UK
5. University of Glasgow, Robertson Centre for Biostatistics, Institute of Health and Wellbeing, Glasgow UK

**Additional Table S1.**
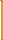
**Previous Studies on Prediction of MTX Response in RA**

| **Study** | **Marker(s)** | **# of Patients** | **Source** | **Performance** | **PMID** |
| --- | --- | --- | --- | --- | --- |
| Seitz et al., 2003 | IL-1ra/IL-1beta ratio | 50 | PBMC | Mixed | 12508386 |
| Hoesktra et al., 2003 | Prior GI events, body mass index, sex, use of NSAIDs, and creatinine clearance | 411 | None | Multivariate analysis, p <0.01 | 12695153 |
| Takatori et al., 2006 | ABCB1 C3435T polymorphism | 124 | PBMC | OR = 8.91, p = 0.001 for NR | 17181924 |
| Parker et al., 2007 | NFkB-regulated genes | 60 | PBMC | No predictive ability | 17696278 |
| Wessels et al., 2007 | Sex, rheumatoid factor and smoking status, the DAS, and 4 polymorphisms in the AMPD1, ATIC, ITPA, and MTHFD1 genes | 186 | PBMC | AUC=0.79-0.85 | 17530705 |
| Aletaha et al., 2007 | SDAI scores (baseline through 24 weeks) | 462 | None | AUC=0.62 (baseline)-0.88 (24 weeks) | 17907167 |
| Hilder et al., 2009 | Baseline median HAQ | 309 | None | Poor predictive ability  AUC=0.59-0.63 | 18292102 |
| Stuhlmuller et al., 2010 | CD11c expression | 34 | Monocytes | No predictive ability | 20032971 |
| Saevarsdottir et al., 2011 | Age, female gender, smoking status | 487 | None | Correlations, no predictions | 21149498 |
| Bugatti et al., 2012 | CXCL13 | 161 | Serum | No predictive ability | 22336440 |
| Owen et al., 2012 | SNPs in ATIC and SLC19A1 genes | 309 | Whole blood | OR = 1.6-1.7 for non-response | 22450926 |
| deRotte et al., 2013 | Erythrocyte folate | 102 | Serum | Linear regression p=0.021 | 24166792 |
| Dhir et al., 2013 | Myeloid progenitor inhibitory factor-1 | 46 | Serum | AUC=0.68  Sensitivity=56%  Specificity=81% | 24455421 |
| Owen et al., 2013 | MTHFR gene SNPs (C677T (rs1801133) and A1298C (rs1801131) | 309 | Whole blood | No association with response | 21931346 |
| Ponchel et al., 2014 | T-cell subsets | 70 | PBMC | NPV = 76%  PPV = 66%  Specificity = 62%  Sensitivity = 79% | 23989988 |
| Chara et al., 2015 | Monocyte numbers/subsets | 52 | PBMC | AUC=0.83-0.94  Sensitivity=77-88%  Specificity=90-100% | 25592233 |
| Ally et al., 2015 | Anti-citrullinated peptide antibodies and cytokines | 140 | PBMC | No predictive ability | 26021985 |
| Tan et al., 2016 | Serum haptoglobin | 69 | PBMCs | AUC=0.76  Sensitivity=74%  Specificity=71% | 24863583 |

**Additional Table S2. Patient characteristics – Discovery cohort**

|  |  | Baseline | |  | 6 months | |  |  |  |
| --- | --- | --- | --- | --- | --- | --- | --- | --- | --- |
|  |  |  |  | *P* |  |  | *P* | Healthy |  |
|  |  |  |  |  |  |  |  |  |  |
|  |  | Non-responder | Responder |  | Non-responder | Responder |  | control |  |
|  |  | (n=4) | (n=4) | value | (n=4) | (n=4) | value | (n=8) |  |
|  | Age – years | 55± 6·1 | 55±19·7 | >0·99 | - | - | - | 52±13·3 |  |
|  | Males – no. (%) | 1 (25) | 1 (25) | 1 | - | - | - | 3 (38) |  |
|  | Caucasian – no. (%) | 4 (100) | 4 (100) | - | - | - | - | 8 (100) |  |
|  | Body mass index – kg/m^2^ | 29·5± 0·96^$^ | 25·0± 4·88 | 0·19 | - | - | - | - |  |
|  | Patient global assessment | 54·3±33·5 | 39·3±30·2 | 0·53 | 54·5±20·0 | 9·3±6·2 | 0·029 | - |  |
|  | (VAS, 0-100 mm) |  |  |  |  |  |  |  |  |
|  | Physician global assessment | 55±29·7 | 38·5±17·8 | 0·38 | 32·5±20·2 | 8·8±7·0 | 0·068 | - |  |
|  | (VAS, 0-100 mm) |  |  |  |  |  |  |  |  |
|  | Number of swollen joints | 11·3±5·3 | 4·8±3·9 | 0·09 | 15±10·7 | 2·0±2·8 | 0·057 | - |  |
|  | (0-28) |  |  |  |  |  |  |  |  |
|  | Number of tender joints (0- | 10·5±7·7 | 4·8±6·4 | 0·2 | 11·25±10·6 | 0·5±1·0 | 0·029 | - |  |
|  | 28) |  |  |  |  |  |  |  |  |
|  | CDAI | 32·7±5·2 | 17·3±9·6 | 0·06 | 35·0±21·2 | 4·3±3·7 | 0·03 | - |  |
|  | DAS28-CRP | 5·1±0·2 | 4.2±0.8 | 0·1 | 5.2±1.4 | 2.2±0.6 | 0.03 | - |  |
|  | DAS28-ESR | 5·2±0.9^$^ | 4.7±0.8 | 0·6 | 5·3±1·3 | 2·8±0·7 | 0·03 | - |  |
|  | RF (IU/ml) | 35·4±25·6 | 321±140^$^ | 0·06 | - | - | - | - |  |
|  | CCP (U/ml) | 10·3±7·2 | 340±0^$^ | 0·06 | - | - | - | - |  |
|  | C-reactive protein (mg/liter) | 8·6±5·3 | 28±19·2 | 0·1 | 23.8±43.5 | 6.9±7.1 | 0·5 | - |  |
|  | Erythrocyte sedimentation | 24±18.3^$^ | 43.8±12.8 | 0·2 | 17·8±18·2 | 27·8±18·2 | 0·17 | - |  |
|  | rate (mm/hour) |  |  |  |  |  |  |  |  |
|  | MTX dosage, mg/week | 17.5±7.1 | 14.4±5.1 | 0.84 | - | - | - | - |  |
|  | Current smoker – no. (%) | 2 (50) | 1 (25) | - | - | - | - | - |  |
|  | Previous smoker – no. (%) | 1 (25) | 1 (25) | - | - | - | - | - |  |
|  | Non-smoker – no. (%) | 1 (25) | 2 (50) | - | - | - | - | - |  |
|  |  |  |  |  |  |  |  |  |  |

We used the Fisher exact unconditional test to assess differences in proportions between the two groups. To examine differences in continuous variables between the two groups, we used the independent samples t-test or the Mann-Whitney U-test (depending on distribution of data).

^$^ n=3

**Additional Table S3. Patient characteristics – Test cohort**

|  | Baseline | |  | 6 months | |  |  |  |
| --- | --- | --- | --- | --- | --- | --- | --- | --- |
|  |  |  | *P* value |  |  | *P* value | Healthy |  |
|  |  |  |  |  |  |  |  |  |
|  | Non-Responder | Responder |  | Non-Responder | Responder |  | control |  |
|  | (n=29) | (n=30) |  | (n=29) | (n=30) |  | (n=30) |  |
| Age – years | 58±14·5 | 54±13·2 | 0·26 | - | - | - | 45±15·4 |  |
| Males – no. (%) | 10 (33) | 13 (43) | 0·6 | - | - | - | 11 (37) |  |
| Caucasian – no. (%) | 30 (100) | 28 (97) ^$^ | - | - | - | - | - |  |
| Body mass index – kg/m^2^ | 28·3± 5·4 | 27·4± 4·6^$$^ | 0·48 | - | - | - | - |  |
| Patient global assessment | 48±30·2 | 62±23·0 | 0·05 | 64±23·2 | 11±12·9 | <0·0001 | - |  |
| (VAS, 0-100 mm) |  |  |  |  |  |  |  |  |
| ^€^Physician global | 46±22·7 | 54±21·0 | 0·19 | 39±6·4 | 6·4±6·1 | <0·0001 | - |  |
| assessment (VAS, 0-100 |  |  |  |  |  |  |  |  |
| mm) |  |  |  |  |  |  |  |  |
| Number of swollen joints | 5·8±3·7 | 8·3±4·3 | 0·006 | 6·0±5·2 | 0·2±0·48 | <0·0001 | - |  |
| (0-28) |  |  |  |  |  |  |  |  |
| Number of tender joints | 8·4±6·2 | 7·9±5·2 | 0·97 | 11·6±7·7 | 0·4±0·72 | <0·0001 | - |  |
| (0-28) |  |  |  |  |  |  |  |  |
| ^€^CDAI | 23·6±10·9 | 27·8±9·8 | 0·13 | 27·9±12·6 | 2·3±2·2 | <0·0001 | - |  |
| DAS28-CRP | 4·7±0·9 | 5·1±0·9 | 0·26 | 5·1±0·9 | 1·9±0·5 | <0·0001 | - |  |
| ^#^DAS28-ESR | 5·2±0·8 | 5·2±1·0 | 0·99 | 5·3±0·8 | 1·7±0·45 | <0·0001 | - |  |
| ^§^RF (IU/ml) | 196±244 | 138±155 | 0·48 | - | - | - | - |  |
| ^¢^CCP (U/ml) | 244±201 | 314±798 | 0·25 | - | - | - | - |  |
| C-reactive protein | 23.2±32.9 | 22.7±29.8 | 0·85 | 13.2±15.8 | 4.8±5·7 | 0·005 | - |  |
| (mg/liter) |  |  |  |  |  |  |  |  |
| ^§^Erythrocyte | 35±19·8 | 22·6±16·2 | 0·02 | 23±18·6 | 8·5±5·6 | 0·0004 | - |  |
| sedimentation rate |  |  |  |  |  |  |  |  |
| (mm/hour) |  |  |  |  |  |  |  |  |
| ^∞^Whole Blood cell count | 8·4±2·2 | 7·5±1·7 | 0·09 | 7·6±2·4 | 6·5±1·7 | 0·07 | - |  |
| ^∞^Neutrophils | 5·6±1·8 | 5·0±1·5 | 0·36 | 5·0±1·8 | 4·2±1·4 | 0·10 | - |  |
| ^∞^Lymphocytes | 1·9±0·59 | 1·7±0·78 | 0·09 | 1·8±0·76 | 1·7±0·95 | 0·31 | - |  |
| ^∞^Monocytes | 0·63±0·16 | 0·59±0·22 | 0·50 | 0·59±0·45 | 0·52±0·13 | 0·38 | - |  |
| ^∞^Eosinophil | 0·18±0·14 | 0·19±0·13 | 0·55 | 0·19±0·15 | 0·17±0·12 | 0·89 | - |  |
| ^∞^Platelets | 332±107 | 307±86 | 0·34 | 299±103 | 270±79 | 0·25 | - |  |

| MTX dosage, mg/week | 20±4.4 | 15±4.9 | 0.002 | - | - | - | - |
| --- | --- | --- | --- | --- | --- | --- | --- |
| Current smoker – no. (%) | 10 (33) | 4 (14) | - | - | - | - | - |
| Previous smoker – no. | 10 (33) | 9 (31) | - | - | - | - | - |
| (%) |  |  |  |  |  |  |  |
| Non-smoker – no. (%) | 10 (33) | 16 (55) | - | - | - | - | - |
|  |  |  |  |  |  |  |  |

We used the Fisher exact unconditional test to assess differences in proportions between the two groups. To examine differences in continuous variables between the two groups, we used the independent samples t-test or the Mann-Whitney U-test (depending on distribution of data).

^$^ One patient “other” (non-white, non-South East Asian, non-Indian Sub-Continent, Non-Afro-Caribbean), one patient did not give an answer.

^$$^ n= 25 in responders for BMI

^€^ Baseline - n = 28 non-R, n= 30 R; 6m - n = 30 non-R, n= 29

^#^ Baseline - n = 19 non-R, n= 23 R; 6m - n = 19 non-R, n= 22 ^§^ Baseline - n = 13 non-R, n= 23 R

^¢^ Baseline - n = 26 non-R, n= 29 R

^∞^ Baseline - n = 29 non-R, n= 27 R; 6m - n = 28 non-R, n= 25

**Additional Table S4. Patient characteristics – Blinded validation cohort**

|  |  | Baseline | |  | 6 months | |  |  |  |
| --- | --- | --- | --- | --- | --- | --- | --- | --- | --- |
|  |  |  |  | *P* value |  |  | *P* value |  |  |
|  |  | Non-Responder | Responder |  | Non-Responder | Responder |  |  |  |
|  |  | (n=10) | (n=9) |  | (n=10) | (n=9) |  |  |  |
|  | Age – years | 54±13.8 | 68±11·3 | 0·03 | - | - | - |  |  |
|  | Males – no. (%) | 4 (40) | 2 (22) | 0·6 | - | - | - |  |  |
|  | Caucasian – no. (%) | 9(90) ^$^ | 9 (100) | - | - | - | - |  |  |
|  | ^$$^Body mass index – kg/m^2^ | 25·6± 2·92 | 27·6± 4·89 | 0·42 | - | - | - |  |  |
|  | ^€^Patient global assessment | 49±14·5 | 45±21·9 | 0·7 | 47±12·1 | 12±11·8 | <0·0001 |  |  |
|  | (VAS, 0-100 mm) |  |  |  |  |  |  |  |  |
|  | Physician global assessment | 52±23·3 | 63±27·5 | 0·4 | 60±13·3 | 16±12·6 | <0·0001 |  |  |
|  | (VAS, 0-100 mm) |  |  |  |  |  |  |  |  |
|  | Number of swollen joints (0-28) | 10·3±4·7 | 8·4±6·1 | 0·5 | 8·2±5·0 | 1·4±2·0 | 0·002 |  |  |
|  | Number of tender joints (0-28) | 11·9±4·5 | 15·6±5·9 | 0·11 | 12·4±4·6 | 0·8±1·3 | <0·0001 |  |  |
|  | CDAI | 32·3±8·1 | 34·3±11·5 | 0·7 | 31·3±9·5 | 4·9±3·8 | <0·0001 |  |  |
|  | DAS28-CRP | 5·5±0·7 | 5·5±0·8 | 0·9 | 5·5±0·9 | 2·4±0·5 | <0·0001 |  |  |
|  | ^€^DAS28-ESR | 5·9±0·9 | 5·9±1·0 | 0·98 | 5·7±1·0 | 2.9±0·6 | 0·0002 |  |  |
|  | ^#^CCP (U/ml) | 213±252 | 109±207 | 0·2 | - | - | - |  |  |
|  | C-reactive protein (mg/liter) | 26·5±28·4 | 16·4±8·8 | 0·32 | 26·0±34·9 | 5·1±3·8 | 0·03 |  |  |
|  | ^€^Erythrocyte sedimentation rate | 39·7±23·9 | 30·3±27·8 | 0·4 | 26·5±21·3 | 17·5±8·8 | 0·2 |  |  |
|  | (mm/hour) |  |  |  |  |  |  |  |  |
|  | ^§^Whole Blood cell count | 8·5±1·6 | 7·8±1·3 | 0·3 | 8·9±3·6 | 6·0±0·65 | 0·06 |  |  |
|  | ^§^Neutrophils | 5·5±1·0 | 5·5±1·5 | 0·9 | 6·1±3·1 | 4·4±1·1 | 0·38 |  |  |
|  | ^§^Lymphocytes | 2·0±0·57 | 1·5±0·78 | 0·1 | 1·9±0·5 | 0·95±0·32 | 0·002 |  |  |
|  | ^§^Monocytes | 0·72±0·26 | 0·57±0·14 | 0·15 | 0·71±0·36 | 0·43±0·15 | 0·24 |  |  |
|  | ^§^Eosinophil | 0·23±0·1 | 0·29±0·18 | 0·36 | 0·16±0·08 | 0·17±0·12 | 0·93 |  |  |
|  | ^§^Platelets | 367±84 | 363±121 | 0·93 | 326±66 | 281±89 | 0·65 |  |  |
|  | MTX dosage, mg/week | 15±5.4 | 15±5.3 | 0.86 | - | - | - | - |  |
|  | Current smoker – no. (%) | 5 (50) | 2 (22) | - | - | - | - |  |  |
|  | Previous smoker – no. (%) | 3 (30) | 5 (56) | - | - | - | - |  |  |
|  | Non-smoker – no. (%) | 2 (20) | 2 (22) | - | - | - | - |  |  |
|  |  |  |  |  |  |  |  |  |  |

We used the Fisher exact unconditional test to assess differences in proportions between the two groups. To examine differences in continuous variables between the two groups, we used the independent samples t-test or the Mann-Whitney U-test (depending on distribution of data).

^$^ One patient “other” (non-white, non-South East Asian, non-Indian Sub-Continent, Non-Afro-Caribbean)

^$$^ Baseline - n= 9 non-R; n= 8 R

^€^ Baseline - n = 10 non-R, n= 6 R; 6m - n = 10 non-R, n= 6

^#^ Baseline - n = 8 non-R, n= 8 R

^§^ Baseline - n = 10 non-R, n= 8 R; 6m - n = 9 non-R, n= 5 R

**Additional Methods**

**Statistical analysis used to generate the CCS**

A LIMMA linear model on the background corrected normalized and log2 transformed data from the array was used to examine the significant CCS markers between RA patients (responder [R] and non-responder [NR]) and Healthy controls (HC). Significance was based on the adjusted p-value (False Discovery Rate [FDR] correction) and Epigenetic Ratios (ER). The ER is the ratio of signature in one sample compared to another, this indicates which of the samples has the potential for forming a chromosome conformation signature. To reduce the number of significant markers we used a stepwise procedure of binary clustering during which the ER of each identified marker was converted to a binary score +1, -1, or 0, this was performed relative to constitutive chromosome conformations, which act as positive controls. Conformations present in NR patients and absent in R patients, were assigned a score of +1, conformations present in R patients and absent in NR patients, were assigned a score of -1 and non-informative non-stratifying conformations received a score of 0. Additional binary filters were applied to markers by ensuring the conformations pattern were specific to the difference between R and NR. This involved the comparison via hierarchical clustering (using Manhattan distance measure with complete linkage agglomeration) of the CCS pattern in the HC vs. R and HC vs. NR. The resultant principle CCS candidate markers from the array screen were then translated onto the EpiSwitch PCR platform for further refinement.

To refine and validate the CCS markers examined in the EpiSwitch PCR analysis**,** we applied a Fisher's exact test for independence using the EpiSwitch binary scores (p < 0.1). Subsequent stepwise refinement of the markers was achieved by building logistic regression models in the WEKA classification platform using five-fold cross validation to score the discerning power of each marker. This was repeated 10 times by random data re-sampling of the initial dataset to generate ten different start points for model generation. The best markers were selected based on the number of times the marker was used by the resampled logistic models to classify the samples.

The accuracy and robustness of the five marker CCS logistic classifying model, was tested by jack-knifing the data set 250 times, generating 250 start points and splitting the data into training and test sets randomly each time using five-fold cross validation. Model performance was analyzed by means of sensitivity (true positive rate), specificity (true negative rate), and using the area under the receiver operating characteristic curve (AUC), which is a combined indicator of sensitivity and specificity, equal to the probability that a classifier will rank a randomly chosen positive instance higher than a randomly chosen negative one. The average model accuracy statistics were adjusted for population response/non-response to MTX (1) using Bayes prevalence theorem (2).

Factor analysis of mixed data (FAMD) was used to evaluate the similarities between individuals taking into account mixed variables, both categorical (class variable) and numerical (EpiSwitch binary scores), to study the relationships between all the variables.

Data analysis was performed in R (language and environment for statistical computing). This included a Stats package for Chi-square test and GLM, ROCR package for ROC curves from WEKA odds probabilities, gplot & stats package for heatmaps. FactorMiner package was used for PCA and Factor plots. WEKA was used for attribute reduction, data randomization and re-sampling, logistic model classifier, AUC calculations and model accuracy calculations.

**Details of assays used to generate the CCS.**

High order chromatin structures were fixed with formaldehyde, extracted, digested with TaqI, and ligated in conditions to maximize intramolecular ligation. Finally, samples were treated with proteinase K. **EpiSwitch™ microarray:** EpiSwitch™ microarray hybridization was performed using the custom Agilent 8x60K array using the Agilent system, following the manufacturer's instructions (Agilent). Each array contains 55,088 probe spots, representing 13,322 potential chromosomal interactions (in quadruplicate) predicted by the EpiSwitch™ pattern recognition software, plus EpiSwitch™ and Agilent controls. Briefly, 1 µg of EpiSwitch™ template was labeled using the Agilent SureTag labeling kit. Processing of labeled DNA was performed. Array analysis was performed immediately after washing using the Agilent scanner and software. In order to compare all the experiments, the data was background corrected and normalized. Since each spot in the array is present in quadruplicate, the median of the four spots of each probe in the array was calculated and its log2 transformed value was used for further analysis. The coefficient of variation and *p*-value was calculated for each probe replicate. **EpiSwitch™ PCR detection:** Oligonucleotides were tested on control templates to confirm that each primer set was working correctly. In line with Royal Forensic Protocol for PCR detection the final nested PCR was performed on each sample in triplicates. This procedure permitted the detection of limited copy-number templates with higher accuracy (3). All PCR amplified products were monitored on the LabChip® GX from Perkin Elmer, using the LabChip DNA 1K Version2 kit (Perkin Elmer) and internal DNA marker was loaded on the DNA chip according to the manufacturer’s protocol using fluorescent dyes. Fluorescence was detected by laser and electropherogram read-outs translated into a simulated band on gel picture using the instrument software. Threshold of detection for the instrument was set by the manufacturer from 30 fluorescence units and above.

**Additional Table S5. Selected genes for EpiSwitch Array**

|  |  | **Number of** |
| --- | --- | --- |
|  |  | **identified** |
| **GENE** | **Description** | **EpiSwitch sites** |
|  |  |  |
| ABCB1 | ATP-binding cassette, sub-family B (MDR/TAP), member 1 | 56 |
| ABCG2 | ATP-binding cassette, sub-family G (WHITE), member 2 | 84 |
| ADORA2A | Adenosine A2a receptor | 72 |
| AFF3 | AF4/FMR2 family, member 3 | 140 |
| AMPD1 | Adenosine monophosphate deaminase 1 | 24 |
| ApoE | Apolipoprotein E | 96 |
| ATIC | 5-aminoimidazole-4-carboxamide ribonucleotide formyltransferase/IMP cyclohydrolase | 32 |
| BLK | B lymphoid tyrosine kinase | 196 |
| BTNL2 | Butyrophilin-like 2 (MHC class II associated) | 44 |
| C5orf30 | Chromosome 5 open reading frame 30 | 96 |
| CCL2 | Chemokine (C-C motif) ligand 2 | 404 |
| CCL21 | Chemokine (C-C motif) ligand 21 | 28 |
| CCL3 | Chemokine (C-C motif) ligand 3 | 52 |
| CCL5 | Chemokine (C-C motif) ligand 5 | 52 |
| CCR1 | Chemokine (C-C motif) receptor 1 | 172 |
| CCR2 | Chemokine (C-C motif) receptor 2 | 164 |
| CCR6 | Chemokine (C-C motif) receptor 6 | 56 |
| CD28 | Cluster of Differentiation 28 | 132 |
| CD40 | Cluster of Differentiation 40 | 148 |
| CD80 | Cluster of Differentiation 80 | 76 |
| CHI3L1 | Chitinase 3-like 1 (cartilage glycoprotein-39) | 64 |
| CHUK | Conserved helix-loop-helix ubiquitous kinase | 92 |
| CIITA | Class II, major histocompatibility complex, transactivator | 80 |
| CLEC12A | C-type lectin domain family 12, member A | 52 |
| CLEC16A | C-type lectin domain family 16, member A | 108 |
| COL2A1 | Collagen, type II, alpha 1 | 100 |
| CTLA4 | Cytotoxic T-lymphocyte-associated protein 4 | 68 |
| CX3CL1 | Chemokine (C-X3-C motif) ligand 1 | 92 |
| CXCL12 | Chemokine (C-X-C motif) ligand 12 | 80 |
| CXCL13 | Chemokine (C-X-C motif) ligand 13 | 80 |
| CXCL8 | Chemokine (C-X-C motif) ligand 8 | 48 |
| CXCR3 | Chemokine (C-X-C motif) receptor 3 | 72 |
| CXCR4 | Chemokine (C-X-C motif) receptor 4 | 56 |
| DHFR | Dihydrofolate reductase | 72 |
| ESR1 | Estrogen receptor 1 | 140 |
| FCGR2A | Fc fragment of IgG, low affinity IIa, receptor (CD32) | 100 |
| FCGR3B | Fc fragment of IgG, low affinity IIIb, receptor (CD16b) | 192 |
| FCRL3 | Fc receptor-like 3 | 68 |
| FPGS | Folylpolyglutamate synthase | 56 |
| HTR2A | 5-hydroxytryptamine (serotonin) receptor 2A, G protein-coupled | 80 |
| ICAM1 | Intercellular adhesion molecule 1 | 132 |
| ICOS | Inducible T-cell co-stimulator | 200 |
| IFNAR1 | Interferon (alpha, beta and omega) receptor 1 | 80 |
| IFNg | Interferon, gamma | 52 |
| IKBKB | Inhibitor of kappa light polypeptide gene enhancer in B-cells, kinase beta | 128 |
| IL-10 | Interleukin 10 | 48 |
| IL-15 | Interleukin 15 | 76 |
| IL-17A | Interleukin 17A | 32 |
| IL-18 | Interleukin 18 | 64 |
| IL-1a | Interleukin 1 alpha | 196 |
| IL-2 | Interleukin 2 | 44 |
| IL-21R | Interleukin 21 receptor | 60 |
| IL-23 | Interleukin 23 | 56 |
| IL-23R | Interleukin 23 receptor | 104 |
| IL-2RA | Interleukin 2 receptor, alpha | 100 |
| IL-2RB | Interleukin 2 receptor, beta | 72 |
| IL-32 | Interleukin 32 | 44 |
| IL-4 | Interleukin 4 | 32 |
| IL-4R | Interleukin 4 receptor | 76 |
| IL-6 | Interleukin 6 | 48 |
| IL-6ST | Interleukin 6 signal transducer (gp130, oncostatin M receptor) | 72 |
| IL-7 | Interleukin 7 | 72 |
| IL1RN | Interleukin 1 receptor antagonist | 28 |
| IRAK3 | Interleukin-1 receptor-associated kinase 3 | 80 |
| IRF5 | Interferon regulatory factor 5 | 76 |
| ITGA4 | Integrin, alpha 4 (antigen CD49D, alpha 4 subunit of VLA-4 receptor) | 100 |
| ITPA | Inosine triphosphatase (nucleoside triphosphate pyrophosphatase) | 56 |
| JAG1 | Jagged 1 | 84 |
| M-CSF | Colony stimulating factor 1 | 96 |
| MafB | V-maf musculoaponeurotic fibrosarcoma oncogene homolog B | 52 |
| MAL | Mal, T-cell differentiation protein | 68 |
| MEFV | Mediterranean fever | 76 |
| MMP14 | Matrix metallopeptidase 14 | 92 |
| MMP2 | Matrix metallopeptidase 2 | 212 |
| MMP9 | Matrix metallopeptidase 9 | 68 |
| MTHFD1 | Methylenetetrahydrofolate dehydrogenase (NADP+ dependent) 1, methenyltetrahydrofolate cyclohydrolase, formyltetrahydrofolate synthetase | 80 |
| MTHFR | Methylenetetrahydrofolate reductase (NAD(P)H) | 52 |
| MyD88 | Myeloid differentiation primary response gene 88 | 80 |
| NFAT | Nuclear factor of activated T cells | 204 |

| NFATC2IP | Nuclear factor of activated T-cells, cytoplasmic, calcineurin-dependent 2 interacting protein | 84 |
| --- | --- | --- |
| NFKB1 | Nuclear factor of kappa light polypeptide gene enhancer in B-cells 1 | 96 |
| NFKB2 | Nuclear factor of kappa light polypeptide gene enhancer in B-cells 2 (p49/p100) | 64 |
| NFKBIA | Nuclear factor of kappa light polypeptide gene enhancer in B-cells inhibitor, alpha | 88 |
| NFKBIB | Nuclear factor of kappa light polypeptide gene enhancer in B-cells inhibitor, beta | 120 |
| NLRP1 | NLR family, pyrin domain containing 1 | 108 |
| NLRP3 | NLR family, pyrin domain containing 3 | 128 |
| PADI4 | Peptidyl arginine deiminase, type IV | 168 |
| PRDM1 | PR domain containing 1, with ZNF domain | 120 |
| PRKCQ | Protein kinase C, theta | 216 |
| PRKCZ | Protein kinase C, zeta | 184 |
| PSTPIP1 | Proline-serine-threonine phosphatase interacting protein 1 | 96 |
| PTGS2 | Prostaglandin-endoperoxide synthase 2 (prostaglandin G/H synthase and cyclooxygenase) | 52 |
| PTPN22 | Protein tyrosine phosphatase, non-receptor type 22 | 196 |
| PXK | PX domain containing serine/threonine kinase | 296 |
| RBPJ | Recombination signal binding protein for immunoglobulin kappa J region | 296 |
| REL | V-rel reticuloendotheliosis viral oncogene homolog A | 92 |
| RFC-1 | Replication factor C (activator 1) 1, 145kDa | 52 |
| RGMB | RGM domain family, member B | 80 |
| RUNX1 | Runt-related transcription factor 1 | 212 |
| SH2B3 | SH2B adaptor protein 3 | 124 |
| SHMT | Serine hydroxymethyltransferase 1 (soluble) | 68 |
| SLC19A1 | Solute carrier family 19 (folate transporter), member 1 | 76 |
| SPRED2 | Sprouty-related, EVH1 domain containing 2 | 336 |
| STAT4 | Signal transducer and activator of transcription 4 | 128 |
| SUMO1 | SMT3 suppressor of mif two 3 homolog 1 | 132 |
| TAGAP | T-cell activation RhoGTPase activating protein | 92 |
| TLR1 | Toll-like receptor 1 | 204 |
| TLR2 | Toll-like receptor 2 | 52 |
| TLR4 | Toll-like receptor 4 | 52 |
| TNF | Tumor necrosis factor | 68 |
| TNFAIP3 | Tumor necrosis factor, alpha-induced protein 3 | 180 |
| TNFRSF11B | Tumor necrosis factor receptor superfamily, member 11b | 80 |
| TNFRSF13C | Tumor necrosis factor receptor superfamily, member 13C | 52 |
| TNFRSF14 | Tumor necrosis factor receptor superfamily, member 14 | 112 |
| TNFRSF17 | Tumor necrosis factor receptor superfamily, member 17 | 44 |
| TNFRSF1A | Tumor necrosis factor receptor superfamily, member 1A | 72 |
| TNFRSF1B | Tumor necrosis factor receptor superfamily, member 1B | 72 |
| TNFSF11 | Tumor necrosis factor (ligand) superfamily, member 11 | 52 |
| TNFSF13 | Tumor necrosis factor (ligand) superfamily, member 13 | 48 |
| TRAF1 | TNF receptor-associated factor 1 | 120 |
| TRAF6 | TNF receptor-associated factor 6 | 72 |
| TYMS | Thymidylate synthetase | 48 |
| WISP3 | WNT1 inducible signaling pathway protein 3 | 88 |


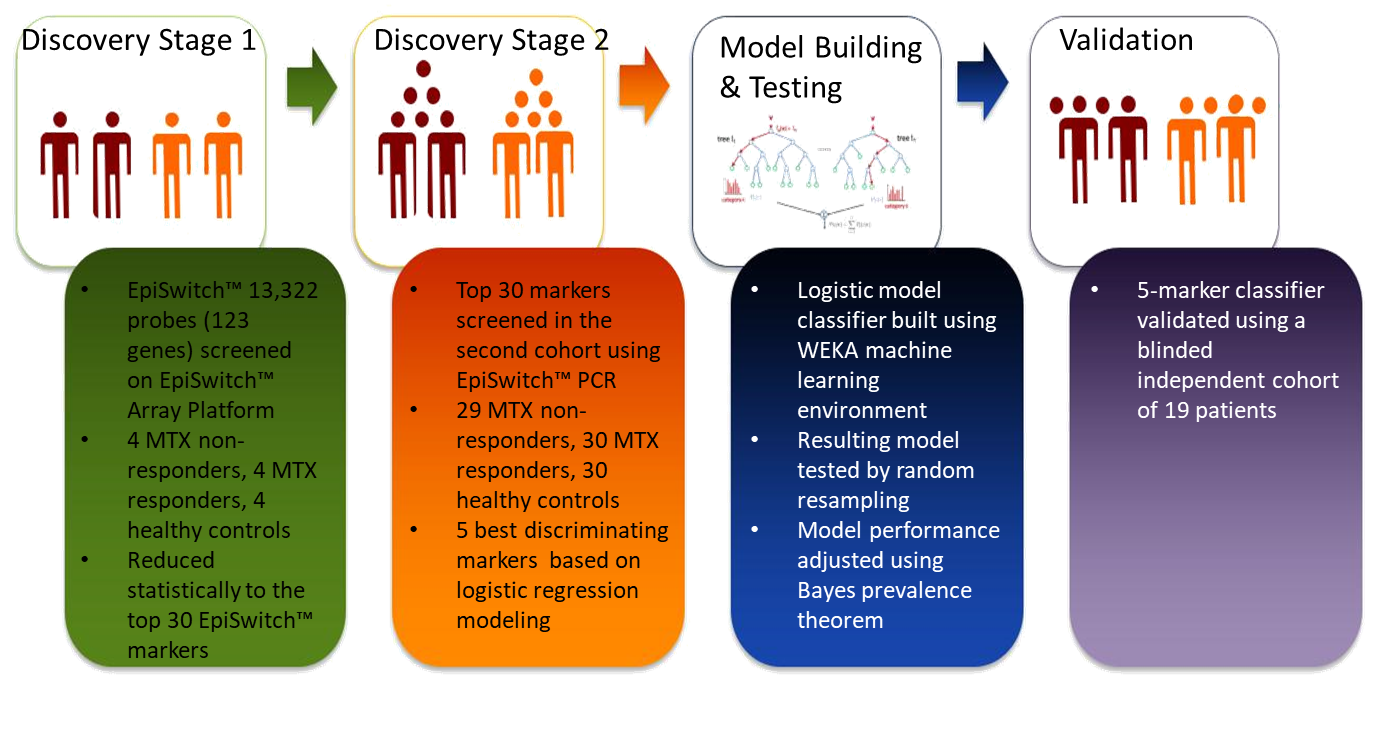


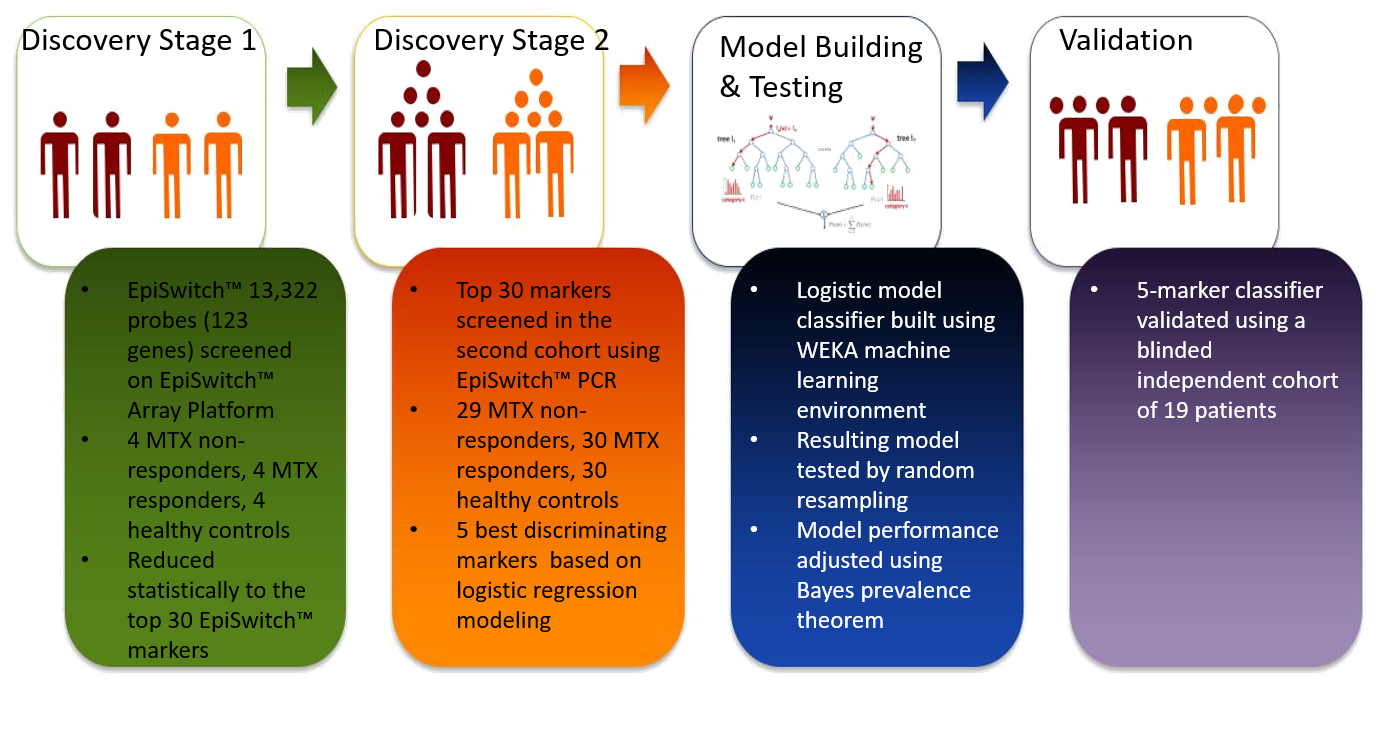


**Additional Figure S1. Design for Discovery and Validation of Epigenetic Stratifying**

**Biomarker Signature for SERA Patients.** Treatment response (responders (R, orange) or non-responders (NR, red)) in patients was confirmed after 6 months of MTX treatment. Epigenetic stratification was based on conditional chromosome conformations screening by EpiSwitch Array, EpiSwitch PCR, model building and testing.


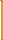

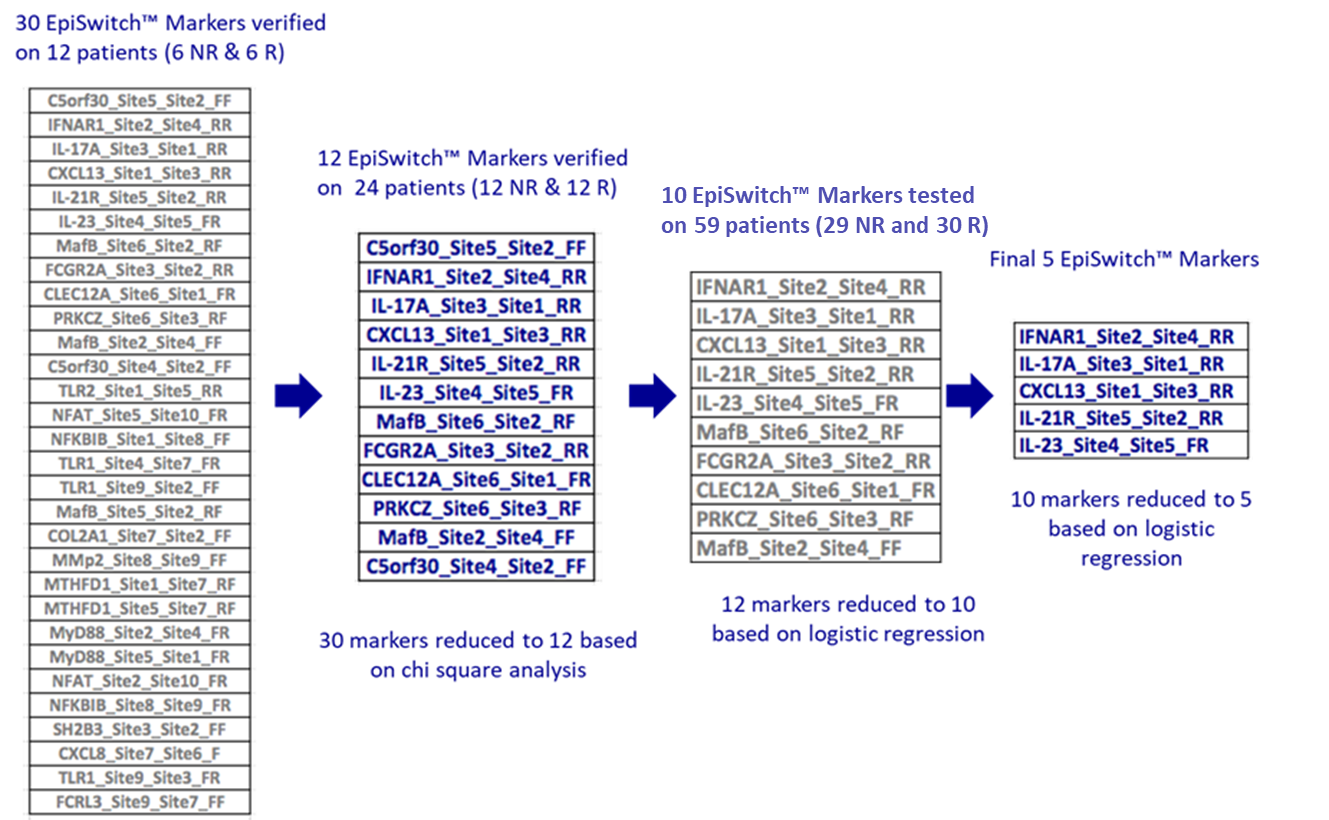


**Additional Table S6.** **Stepwise marker selection.** Selection of the markers translated into EpiSwitch™ PCR platform. The second patient cohort totalled 59 RA patients, 30 MTX-Responders and 29 MTX-Non-Responders. The 30 markers selected for PCR translation and validation were first run in 12 patients, 6 MTX-Responders and 6 MTX-Non-Responders, the most discerning markers were determined by applying a Chi-squared test for independence with Yate’s continuity correction on the binary scores. This reduced the initial 30-marker set selected from the array screen down to 12. The resultant 12 markers were then screened in an additional 12 patients, 6 MTX-Responders and 6 MTX-Non-Responders, increasing the number of patients screened for these 12 markers to 24. The markers were then further reduced to 10 discerning markers in the 24-patient screen by building a logistic regression Model in WEKA (weka-3-6-12) classification platform using 5-fold cross validation to score the discerning power of each marker. The best markers were selected based on the score or number of folds that selected the marker for classification. This was repeated 10 times by random data resampling of the initial data set to generate 10 different start points for model generation. The markers used the most were selected for screening in the remainder of the patient cohort (25 patients, 12 MTX-Responders and 13 MTX-Non-Responders). 10 EpiSwitch™ markers were selected by this collated effort and these were run in all 59 patients. The final marker set was reduced further to 5 EpiSwitch™ markers using the same procedure described on the 59-patient cohort. The final 5 EpiSwitch™ marker set were then used in model testing and validation.

**Additional Table S7**. **65 Selected genes from EpiSwitch Array analysis**

|  |  | **adjusted p** | **Episwitch** |  |  |  |  |
| --- | --- | --- | --- | --- | --- | --- | --- |
| **Gene** | **Probes*** | **value** | **ratio** | **HC_NR_MTX** | **HC_R_MTX** | **NR_R_MTX** | **Association** |
|  |  |  |  |  |  |  |  |
| 19_55449062_55451429_55484960_55486708_RF | 19_55449062_55451429_55484960_55486708_RF | 0.079 | -1.43 | 0 | -1 | -1 | R |
| C5orf30 | C5orf30_Site5_Site2_FF | 0.079 | -1.24 | 1 | -1 | -1 | R |
| CHUK | CHUK_Site7_Site2_RF | 0.079 | -1.33 | 1 | -1 | -1 | R |
| CXCL13 | CXCL13_Site1_Site3_RR | 0.079 | -1.30 | 0 | -1 | -1 | R |
| TLR1 | TLR1_Site4_Site7_FR | 0.079 | -1.43 | 1 | -1 | -1 | R |
| 11_47175706_47180170_47251505_47252468_FR | 11_47175706_47180170_47251505_47252468_FR | 0.083 | -1.21 | 1 | -1 | -1 | R |
| C5orf30 | C5orf30_Site4_Site2_FF | 0.084 | -1.20 | 1 | -1 | -1 | R |
| TLR1 | TLR1_Site9_Site2_FF | 0.087 | -1.38 | 1 | -1 | -1 | R |
| FCRL3 | FCRL3_Site9_Site7_FF | 0.090 | -1.25 | 1 | -1 | -1 | R |
| SH2B3 | SH2B3_Site6_Site5_FF | 0.090 | -1.33 | 1 | -1 | -1 | R |
| 12_69705360_69711928_69799162_69800678_RF | 12_69705360_69711928_69799162_69800678_RF | 0.097 | -1.21 | 1 | -1 | -1 | R |
| IL-23R | IL-23R_Site5_Site8_FF | 0.109 | -1.27 | 1 | -1 | -1 | R |
| CLEC12A | CLEC12A_Site6_Site1_FR | 0.113 | -1.22 | 0 | -1 | -1 | R |
| IL-17A | IL-17A_Site3_Site1_RR | 0.115 | -1.16 | 0 | -1 | -1 | R |
| CXCL8 | CXCL8_Site7_Site6_FR | 0.118 | -1.13 | 0 | **-1** | **-1** | R |
| MyD88 | MyD88_Site5_Site1_FR | 0.130 | -1.18 | 1 | 0 | -1 | R |
| PRDM1 | PRDM1_Site6_Site2_RR | 0.144 | -1.19 | 1 | -1 | -1 | R |
| MMP2 | MMP2_Site8_Site9_FF | 0.146 | -1.21 | 1 | -1 | -1 | R |
| SPRED2 | SPRED2_Site4_Site8_RF | 0.149 | -1.39 | 1 | -1 | -1 | R |
| C5orf30 | C5orf30_Site4_Site8_RF | 0.150 | -1.18 | 1 | -1 | -1 | R |
| 19_10294661_10295285_10370560_10371551_RR | 19_10294661_10295285_10370560_10371551_RR | 0.153 | -1.21 | 1 | -1 | -1 | R |
| TNFRSF13C | TNFRSF13C_Site3_Site6_FF | 0.153 | -1.21 | 1 | -1 | -1 | R |
| IL-23 | IL-23_Site4_Site5_FR | 0.161 | -1.18 | 0 | -1 | -1 | R |
| NFKBIB | NFKBIB_Site8_Site9_FR | 0.168 | -1.23 | 1 | -1 | -1 | R |
| TNFRSF13C | TNFRSF13C_Site1_Site6_FF | 0.169 | -1.12 | 1 | -1 | -1 | R |
| CD28 | CD28_Site5_Site9_RR | 0.172 | -1.14 | 1 | -1 | -1 | R |
| NFKB1 | NFKB1_Site4_Site8_RR | 0.186 | -1.20 | 1 | -1 | -1 | R |
| CHUK | CHUK_Site3_Site5_RF | 0.188 | -1.13 | 1 | -1 | -1 | R |
| TLR1 | TLR1_Site9_Site3_FR | 0.188 | -1.20 | 1 | -1 | -1 | R |
| M-CSF | M-CSF_Site5_Site6_FF | 0.191 | -1.21 | 1 | -1 | -1 | R |
| NFKBIB | NFKBIB_Site1_Site8_FF | 0.192 | -1.13 | 1 | -1 | -1 | R |
| 11_47175706_47180170_47202910_47204016_FF | 11_47175706_47180170_47202910_47204016_FF | 0.192 | -1.21 | 1 | -1 | -1 | R |
| PRDM1 | PRDM1_Site6_Site1_RR | 0.195 | -1.19 | 1 | -1 | -1 | R |
| TNFRSF14 | TNFRSF14_Site4_Site1_RR | 0.082 | 1.53 | 0 | 1 | 1 | NR |
| SH2B3 | SH2B3_Site3_Site2_FF | 0.083 | 1.23 | -1 | 1 | 1 | NR |
| MyD88 | MyD88_Site2_Site4_FR | 0.086 | 1.21 | 0 | 1 | 1 | NR |
| MafB | MafB_Site2_Site4_FF | 0.091 | 1.17 | -1 | 1 | 1 | NR |
| PRKCZ | PRKCZ_Site6_Site3_RF | 0.094 | 1.32 | 0 | **1** | **1** | NR |
| IFNAR1 | IFNAR1_Site2_Site4_RR | 0.094 | 1.23 | -1 | 1 | 1 | NR |
| NFAT | NFAT_Site2_Site10_FR | 0.094 | 1.21 | -1 | 1 | 1 | NR |
| NFAT | NFAT_Site5_Site10_RR | 0.094 | 1.25 | -1 | 1 | 1 | NR |
| MAL | MAL_Site2_Site6_RF | 0.095 | 1.27 | 0 | 1 | 1 | NR |

|  | **Key** | |
| --- | --- | --- |
|  |  |  |
| HC_NR_MTX |  | 1= loop in HC |
|  |  | 0=Not_Relevant |
|  |  | "-1"= loop in NR |
| HC_R_MTX |  | 1= loop in HC |
|  |  | 0=Not_Relevant |
|  |  | "-1"= loop in R |
| NR_R_MTX |  | 1= loop in NR |
|  |  | 0=Not_Relevant |
|  |  | "-1"= loop in R |

* Probes were designed based on 3 dimensional orientation of the chromosomal confirmation sites. Hence, these were either FF (Forward-Forward), FR (Forward-Reverse), RF (Reverse-Forward) or RR (Reverse-Reverse)

| FCGR2A | FCGR2A_Site3_Site6_RR | 0.097 | 1.17 | -1 | 1 | 1 | NR |
| --- | --- | --- | --- | --- | --- | --- | --- |
| IL-32 | IL-32_Site5_Site4_RF | 0.097 | 1.21 | 0 | 1 | 1 | NR |
| MTHFD1 | MTHFD1_Site1_Site7_RF | 0.115 | 1.18 | -1 | 1 | 1 | NR |
| TLR2 | TLR2_Site1_Site5_RR | 0.121 | 1.22 | -1 | 1 | 1 | NR |
| NFAT | NFAT_Site6_Site10_RR | 0.130 | 1.21 | -1 | 1 | 1 | NR |
| ICAM1 | ICAM1_Site4_Site9_FR | 0.131 | 1.18 | -1 | 1 | 1 | NR |
| NFAT | NFAT_Site5_Site10_FR | 0.133 | 1.17 | -1 | 1 | 1 | NR |
| MTHFD1 | MTHFD1_Site5_Site7_RF | 0.145 | 1.16 | -1 | 1 | 1 | NR |
| MTHFR | MTHFR_Site6_Site4_RR | 0.150 | 1.17 | -1 | 1 | 1 | NR |
| ICAM1 | ICAM1_Site4_Site1_FF | 0.151 | 1.14 | -1 | 1 | 1 | NR |
| MTHFD1 | MTHFD1_Site1_Site7_RF | 0.115 | 1.18 | **-1** | **1** | **1** | NR |
| NFAT | NFAT_Site11_Site10_RR | 0.159 | 1.20 | -1 | 1 | 1 | NR |
| NFAT | NFAT_Site10_Site9_RF | 0.161 | 1.20 | -1 | 1 | 1 | NR |
| MafB | MafB_Site5_Site2_RF | 0.167 | 1.16 | -1 | 1 | 1 | NR |
| NFAT | NFAT_Site7_Site10_RR | 0.170 | 1.19 | -1 | 1 | 1 | NR |
| FCGR2A | FCGR2A_Site3_Site7_RR | 0.180 | 1.13 | -1 | 1 | 1 | NR |
| MafB | MafB_Site6_Site2_RF | 0.187 | 1.11 | -1 | 1 | 1 | NR |
| ADORA2A | ADORA2A_Site1_Site7_FR | 0.191 | 1.14 | -1 | 1 | 1 | NR |
| MMP9 | MMP9_Site2_Site3_FR | 0.192 | 1.13 | -1 | 1 | 1 | NR |
| COL2A1 | COL2A1_Site7_Site2_FF | 0.194 | 1.11 | -1 | 1 | 1 | NR |
| TNFRSF1B | TNFRSF1B_Site1_Site7_FR | 0.196 | 1.15 | -1 | 1 | 1 | NR |
| FCGR2A | FCGR2A_Site3_Site2_RR | 0.198 | 1.12 | -1 | 1 | 1 | NR |
| IL-21R | IL-21R_Site5_Site2_RR | 0.199 | 1.13 | 0 | 1 | 1 | NR |
| **30 Selected genes from EpiSwitch Array** |  |  |  |  |  |  |  |
| **Gene** | **EpiSwitch Marker** | **adjusted.p.val** | **Episwitch.rati** | **HC_NR_MTX** | **HC_R_MTX** | **NR_R_MTX** | **Association** |
|  |  |  |  |  |  |  |  |
| C5orf30 | C5orf30_Site4_Site2_FF | 0.084 | -1.20 | 1 | -1 | -1 | R |
| C5orf30 | C5orf30_Site5_Site2_FF | 0.079 | -1.24 | 1 | -1 | -1 | R |
| CLEC12A | CLEC12A_Site6_Site1_FR | 0.113 | -1.22 | 0 | -1 | -1 | R |
| COL2A1 | COL2A1_Site7_Site2_FF | 0.194 | 1.11 | -1 | 1 | 1 | NR |
| CXCL13 | CXCL13_Site1_Site3_RR | 0.079 | -1.30 | 0 | -1 | -1 | R |
| CXCL8 | CXCL8_Site7_Site6_FR | 0.118 | -1.13 | 0 | -1 | -1 | R |
| FCGR2A | FCGR2A_Site3_Site2_RR | 0.198 | 1.12 | -1 | 1 | 1 | NR |
| FCRL3 | FCRL3_Site9_Site7_FF | 0.090 | -1.25 | 1 | -1 | -1 | R |
| IFNAR1 | IFNAR1_Site2_Site4_RR | 0.094 | 1.23 | -1 | 1 | 1 | NR |
| IL-17A | IL-17A_Site3_Site1_RR | 0.115 | -1.16 | 0 | -1 | -1 | R |
| IL-21R | IL-21R_Site5_Site2_RR | 0.199 | 1.13 | 0 | 1 | 1 | NR |
| IL-23 | IL-23_Site4_Site5_FR | 0.161 | -1.18 | 0 | -1 | -1 | R |
| MafB | MafB_Site2_Site4_FF | 0.091 | 1.17 | -1 | 1 | 1 | NR |
| MafB | MafB_Site5_Site2_RF | 0.167 | 1.16 | -1 | 1 | 1 | NR |
| MafB | MafB_Site6_Site2_RF | 0.187 | 1.11 | -1 | 1 | 1 | NR |
| MMP2 | MMP2_Site8_Site9_FF | 0.146 | -1.21 | 1 | -1 | -1 | R |
| MTHFD1 | MTHFD1_Site1_Site7_RF | 0.115 | 1.18 | **-1** | **1** | **1** |  |
| MTHFD1 | MTHFD1_Site5_Site7_RF | 0.145 | 1.16 | -1 | 1 | 1 | NR |
| MyD88 | MyD88_Site2_Site4_FR | 0.086 | 1.21 | 0 | 1 | 1 | NR |
| MyD88 | MyD88_Site5_Site1_FR | 0.130 | -1.18 | 1 | 0 | -1 | R |

| NFAT | NFAT_Site2_Site10_FR | 0.094 | 1.21 | -1 | 1 | 1 | NR |
| --- | --- | --- | --- | --- | --- | --- | --- |
| NFAT | NFAT_Site5_Site10_FR | 0.133 | 1.17 | -1 | 1 | 1 | NR |
| NFKBIB | NFKBIB_Site1_Site8_FF | 0.192 | -1.13 | 1 | -1 | -1 | R |
| NFKBIB | NFKBIB_Site8_Site9_FR | 0.168 | -1.23 | 1 | -1 | -1 | R |
| PRKCZ | PRKCZ_Site6_Site3_RF | 0.094 | 1.32 | 0 | 1 | 1 | NR |
| SH2B3 | SH2B3_Site3_Site2_FF | 0.083 | 1.23 | -1 | 1 | 1 | NR |
| TLR1 | TLR1_Site4_Site7_FR | 0.079 | -1.43 | 1 | -1 | -1 | R |
| TLR1 | TLR1_Site9_Site2_FF | 0.087 | -1.38 | 1 | -1 | -1 | R |
| TLR1 | TLR1_Site9_Site3_FR | 0.188 | -1.20 | 1 | -1 | -1 | R |
| TLR2 | TLR2_Site1_Site5_RR | 0.121 | 1.22 | -1 | 1 | 1 | NR |

**Additional Table S8**

**12 Selected genes from EpiSwitch PCR**

| **Gene** | **EpiSwitch Marker** | **adjusted.p.value** | **Episwitch.ratio** | **HC_NR_MTX** | **HC_R_MTX** |  | **NR_R_MTX** |  | **Association** |
| --- | --- | --- | --- | --- | --- | --- | --- | --- | --- |
| C5orf30 | C5orf30_Site5_Site2_FF | 0.079 | -1.24 | 1 |  | -1 |  | -1 | R |
| IFNAR1 | IFNAR1_Site2_Site4_RR | 0.094 | 1.23 | -1 |  | 1 |  | 1 | NR |
| IL-17A | IL-17A_Site3_Site1_RR | 0.115 | -1.16 | 0 |  | -1 |  | -1 | R |
| CXCL13 | CXCL13_Site1_Site3_RR | 0.079 | -1.30 | 0 |  | -1 |  | -1 | R |
| IL-21R | IL-21R_Site5_Site2_RR | 0.199 | 1.13 | 0 |  | 1 |  | 1 | NR |
| IL-23 | IL-23_Site4_Site5_FR | 0.161 | -1.18 | 0 |  | -1 |  | -1 | R |
| MafB | MafB_Site6_Site2_RF | 0.187 | 1.11 | -1 |  | 1 |  | 1 | NR |
| FCGR2A | FCGR2A_Site3_Site2_RR | 0.198 | 1.12 | -1 |  | 1 |  | 1 | NR |
| CLEC12A | CLEC12A_Site6_Site1_FR | 0.113 | -1.22 | 0 |  | -1 |  | -1 | R |
| PRKCZ | PRKCZ_Site6_Site3_RF | 0.094 | 1.32 | 0 |  | 1 |  | 1 | NR |
| MafB | MafB_Site2_Site4_FF | 0.091 | 1.17 | -1 |  | 1 |  | 1 | NR |
| C5orf30 | C5orf30_Site4_Site2_FF | 0.084 | -1.20 | 1 |  | -1 |  | -1 | R |
| **10 Selected genes from EpiSwitch PCR** | |  |  |  |  |  |  |  |  |
| **Gene** | **EpiSwitch Marker** | **adjusted.p.value** | **Episwitch.ratio** | **HC_NR_MTX** | **HC_R_MTX** |  | **NR_R_MTX** |  | **Association** |
| IFNAR1 | IFNAR1_Site2_Site4_RR | 0.094 | 1.23 | -1 |  | 1 |  | 1 | NR |
| IL-17A | IL-17A_Site3_Site1_RR | 0.115 | -1.16 | 0 |  | -1 |  | -1 | R |
| CXCL13 | CXCL13_Site1_Site3_RR | 0.079 | -1.30 | 0 |  | -1 |  | -1 | R |
| IL-21R | IL-21R_Site5_Site2_RR | 0.199 | 1.13 | 0 |  | 1 |  | 1 | NR |
| IL-23 | IL-23_Site4_Site5_FR | 0.161 | -1.18 | 0 |  | -1 |  | -1 | R |
| MafB | MafB_Site6_Site2_RF | 0.187 | 1.11 | -1 |  | 1 |  | 1 | NR |
| FCGR2A | FCGR2A_Site3_Site2_RR | 0.198 | 1.12 | -1 |  | 1 |  | 1 | NR |
| CLEC12A | CLEC12A_Site6_Site1_FR | 0.113 | -1.22 | 0 |  | -1 |  | -1 | R |
| PRKCZ | PRKCZ_Site6_Site3_RF | 0.094 | 1.32 | 0 |  | 1 |  | 1 | NR |
| MafB | MafB_Site2_Site4_FF | 0.091 | 1.17 | -1 |  | 1 |  | 1 | NR |
| **5 Selected genes from EpiSwitch PCR** | |  |  |  |  |  |  |  |  |
| **Gene** | **EpiSwitch Marker** | **adjusted.p.value** | **Episwitch.ratio** | **HC_NR_MTX** | **HC_R_MTX** |  | **NR_R_MTX** |  | **Association** |
| IFNAR1 | IFNAR1_Site2_Site4_RR | 0.094 | 1.23 | -1 |  | 1 |  | 1 | NR |
| IL-17A | IL-17A_Site3_Site1_RR | 0.115 | -1.16 | 0 |  | -1 |  | -1 | R |
| CXCL13 | CXCL13_Site1_Site3_RR | 0.079 | -1.30 | 0 |  | -1 |  | -1 | R |
| IL-21R | IL-21R_Site5_Site2_RR | 0.199 | 1.13 | 0 |  | 1 |  | 1 | NR |
| IL-23 | IL-23_Site4_Site5_FR | 0.161 | -1.18 | 0 |  | -1 |  | -1 | R |

|  | **Key** | |
| --- | --- | --- |
|  |  |  |
| HC_NR_MTX |  | 1= loop in HC |
|  |  | 0=Not_Relevant |
|  |  | "-1"= loop in NR |
| HC_R_MTX |  | 1= loop in HC |
|  |  | 0=Not_Relevant |
|  |  | "-1"= loop in R |
| NR_R_MTX |  | 1= loop in NR |
|  |  | 0=Not_Relevant |
|  |  | "-1"= loop in R |


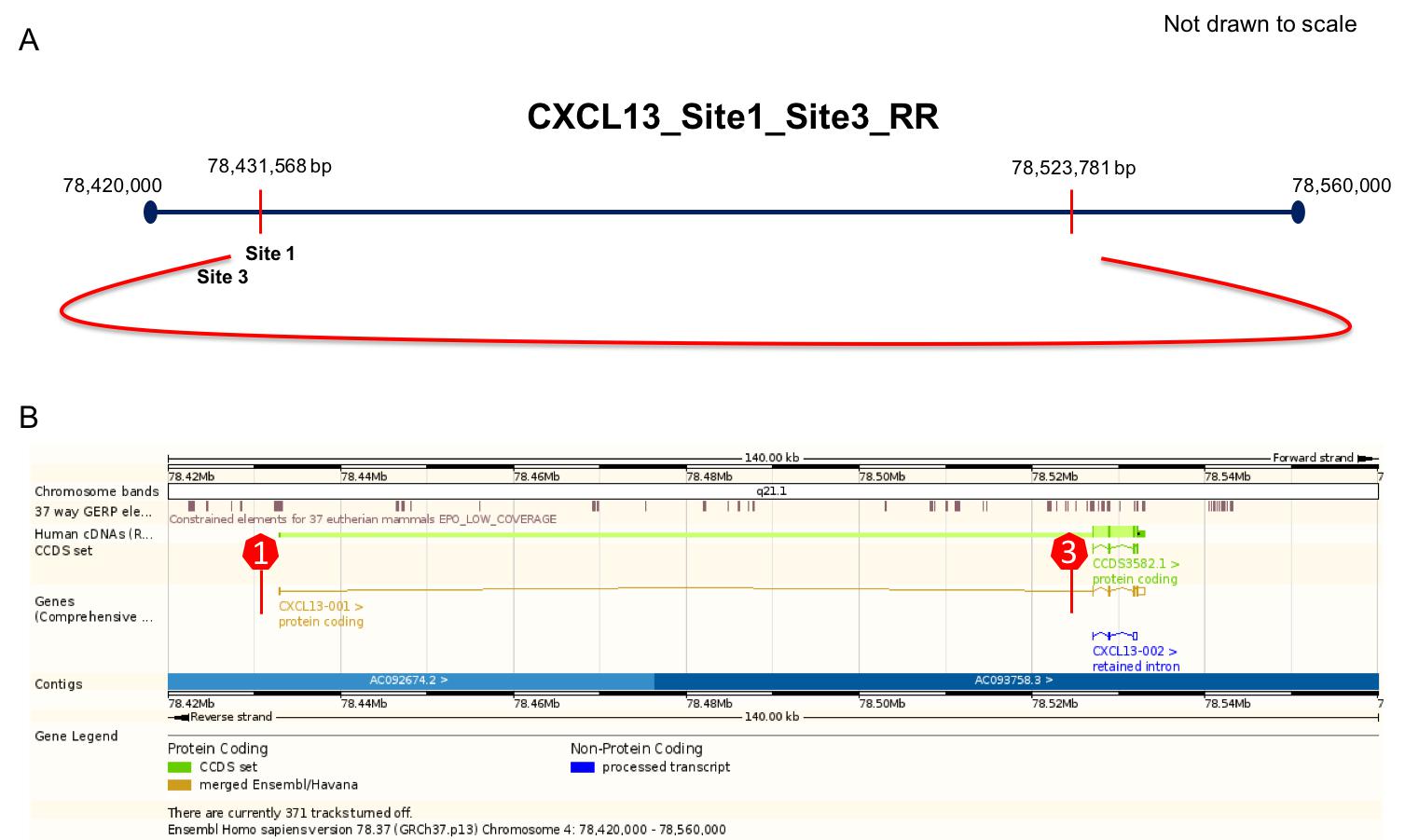


**C**

**
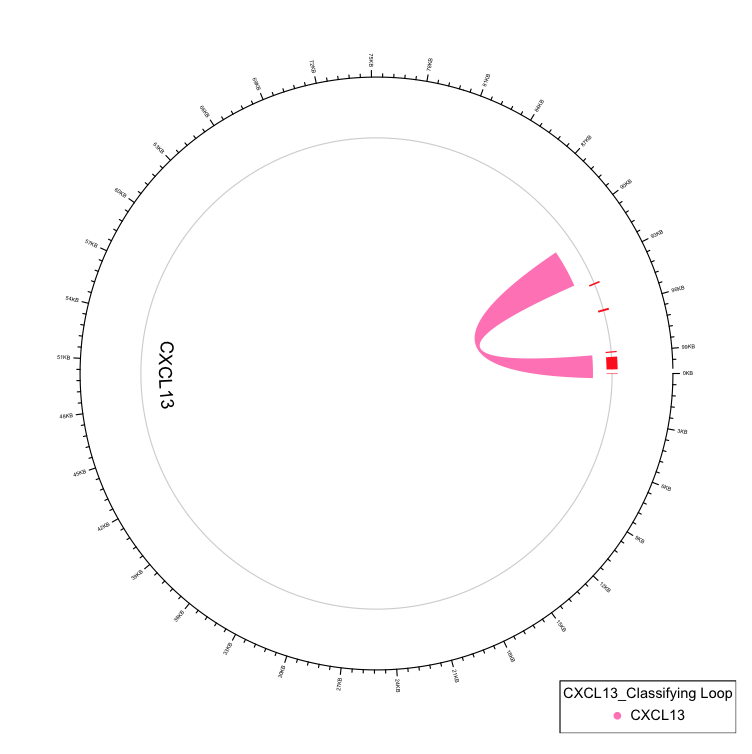
**

**Additional Figure S2. Graphical representation of the genomic co-ordinates of the**

**CXCL13 CCS marker associated with non-response to MTX.** (A) The line

represents the region on chromosome 4 from 78,420,00 to 78,560,000 with the two EpiSwitch co-ordinates (Red) that form the chromosomal loop. Orientation of EpiSwitch site/loop is indicated RR (reverse-reverse). (B) The Ensembl browser view of the CXCL13 gene on chromosome 4 with the EpiSwitch^TM^ sites marked with red symbols.

(C) Circos plot of the CXCL13 CCS marker showing the chromosomal loop (pink).


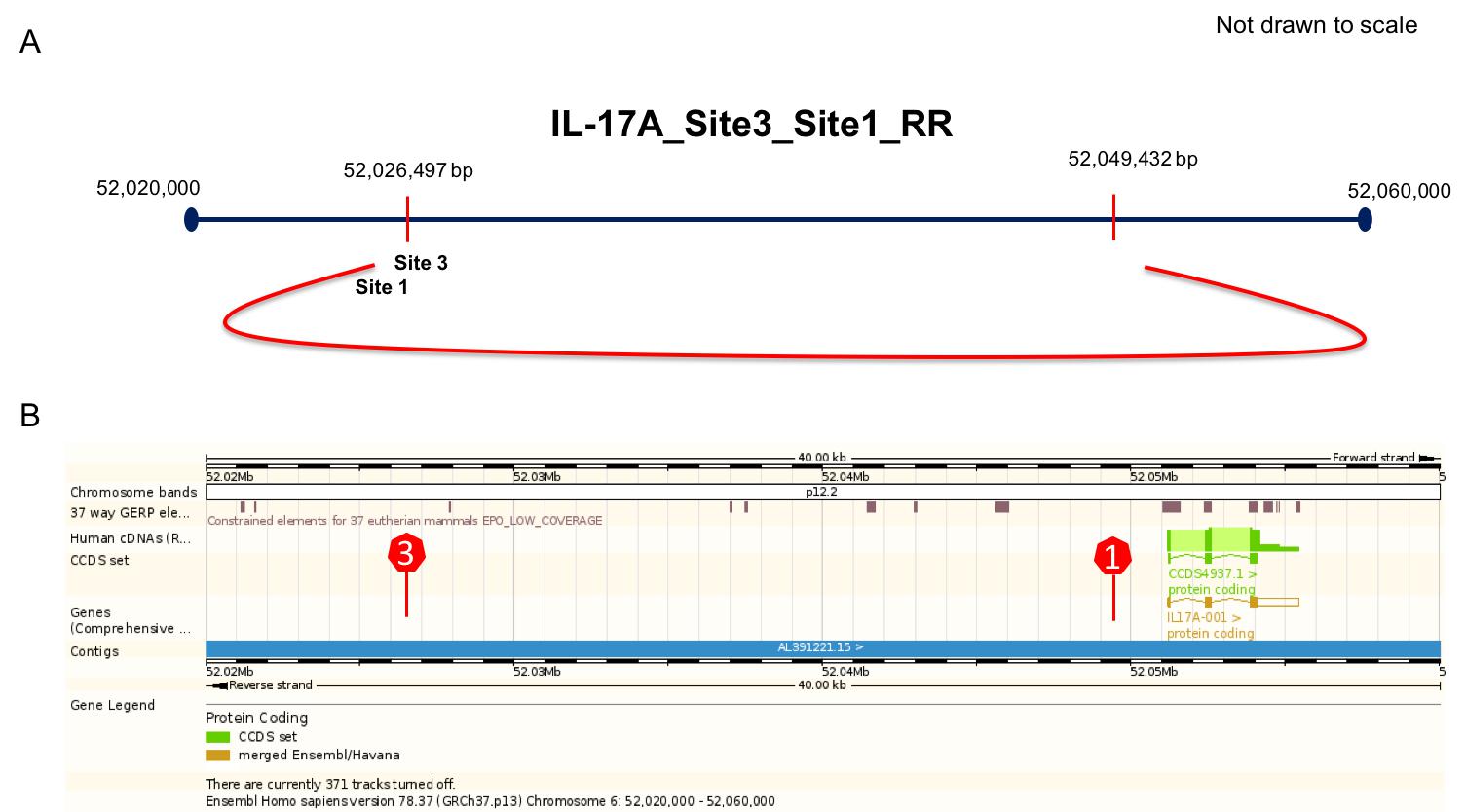


**C**

**
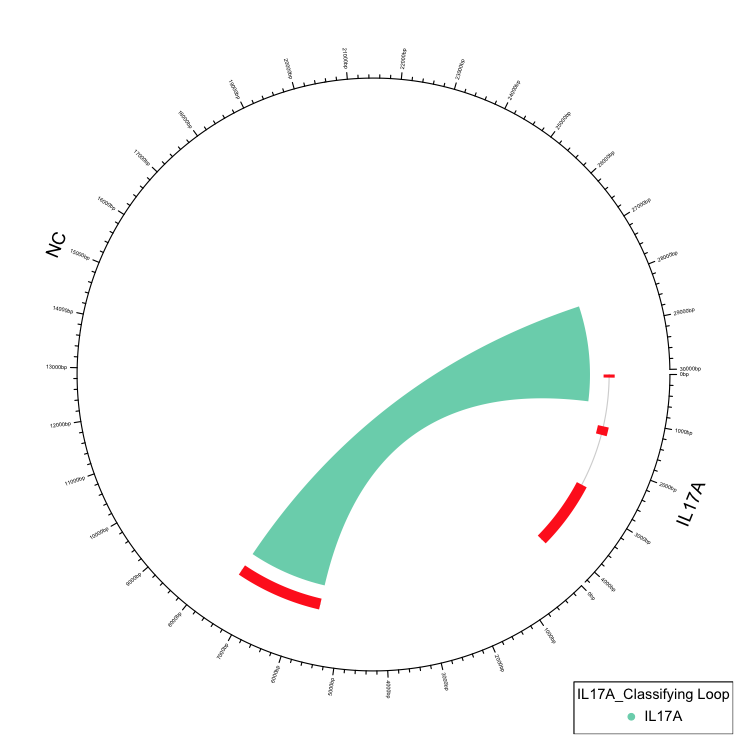
**

**Additional Figure S3. Graphical representation of the genomic co-ordinates of the IL-**

**17A CCS marker associated with non-response to MTX.** (A) The line represents the region on chromosome 6 from 52,020,000 to 52,060,000 with the two EpiSwitch co-ordinates (Red) that form the chromosomal loop. Orientation of EpiSwitch site/loop is indicated by RR (reverse-reverse). (B) The Ensembl browser view of the IL-17A gene on chromosome 6 with the EpiSwitch^TM^ sites marked with red symbols. (C) Circos plot of the IL-17A CCS marker showing the chromosomal loop (green).


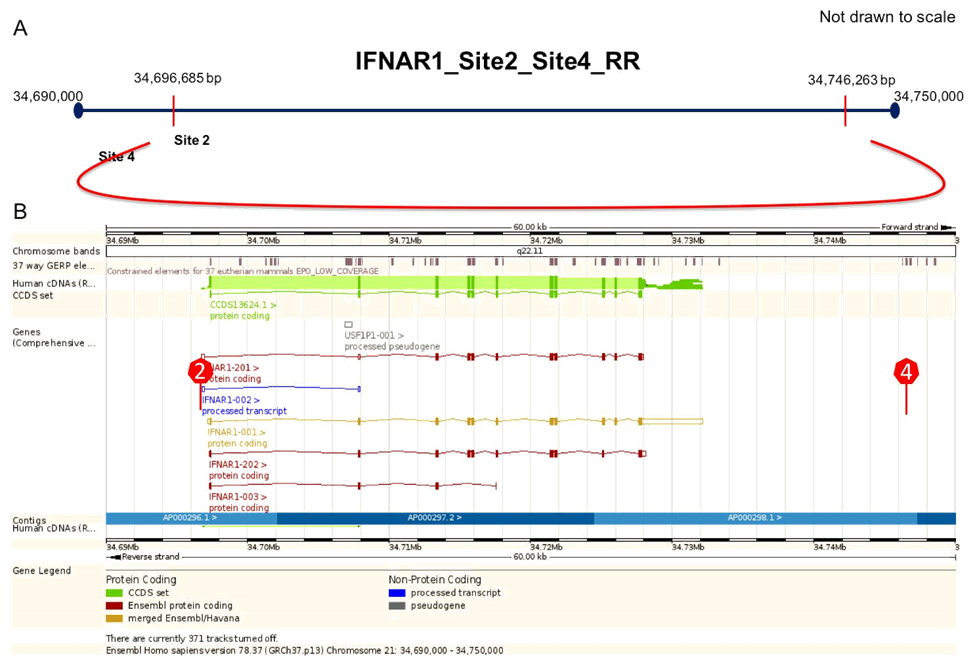

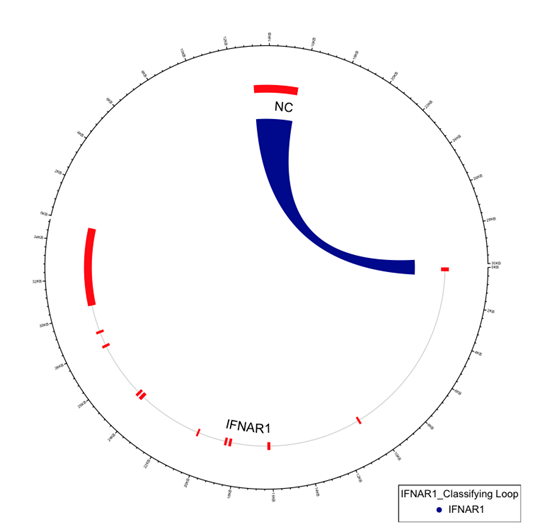


**Additional Figure S4. Graphical representation of the genomic co-ordinates of the**

**IFNAR1 CCS marker associated with non-response to MTX.** (A) The line represents the region on chromosome 21 from 34,690,000 to 34,750,000 with the two EpiSwitch co-ordinates (Red) that form the chromosomal loop. Orientation of EpiSwitch site/loop is indicated by RR (reverse-reverse). (B) The Ensembl browser view of the IFNAR1 gene on chromosome 21 with the EpiSwitch^TM^ sites marked with red symbols. (C) Circos plot of the IFNAR1 CCS marker showing the chromosomal loop (blue).

**
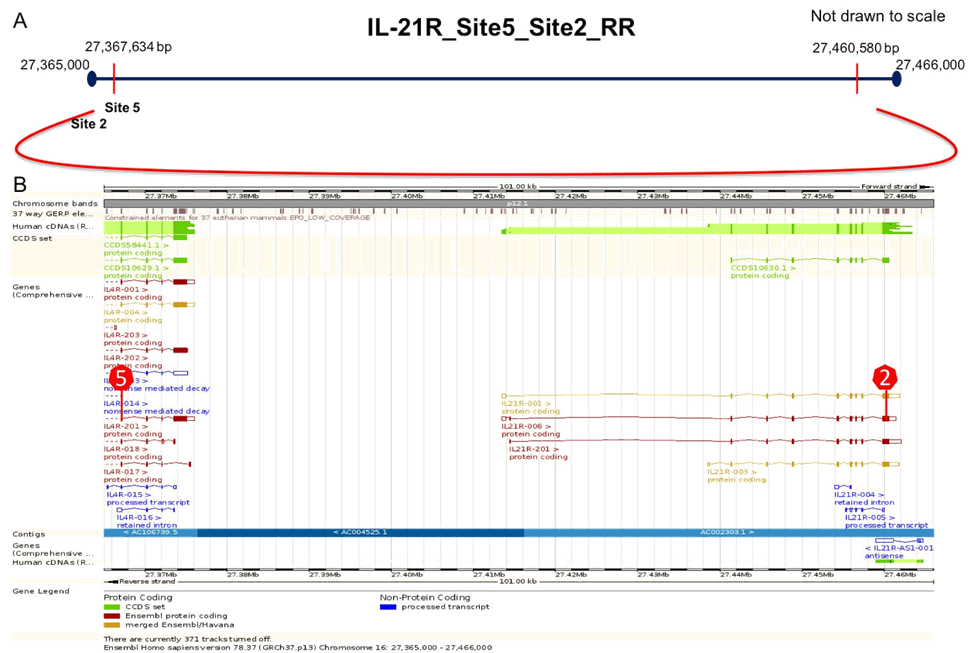
**

**C**

**
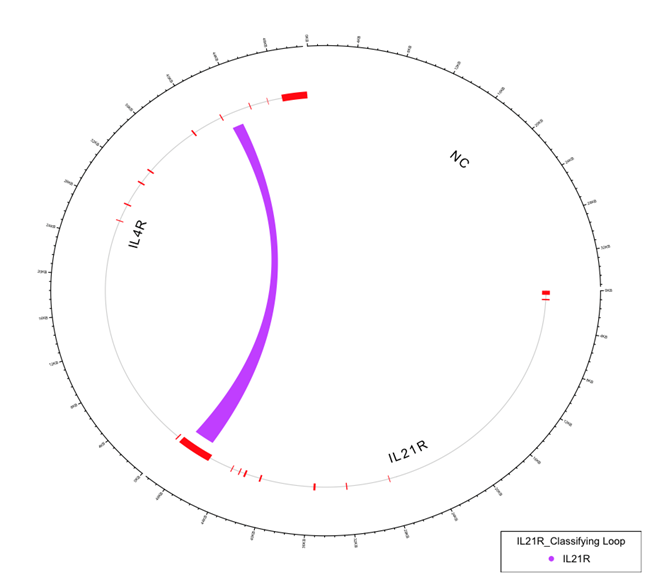
**

**Additional Figure S5. Graphical representation of the genomic co-ordinates of the IL-**

**21R CCS marker associated with non-response to MTX.** (A) The line represents the region on chromosome 16 from 27,365,00 to 27,466,000 with the two EpiSwitch co-ordinates (Red) that form the chromosomal loop. Orientation of EpiSwitch site/loop is indicated by RR (reverse-reverse). (B) The Ensembl browser view of the IFNAR1 gene on chromosome 16 with the EpiSwitch^TM^ sites marked with red symbols. (C) Circos plot of the IL2 CCS marker showing the chromosomal loop (purple).


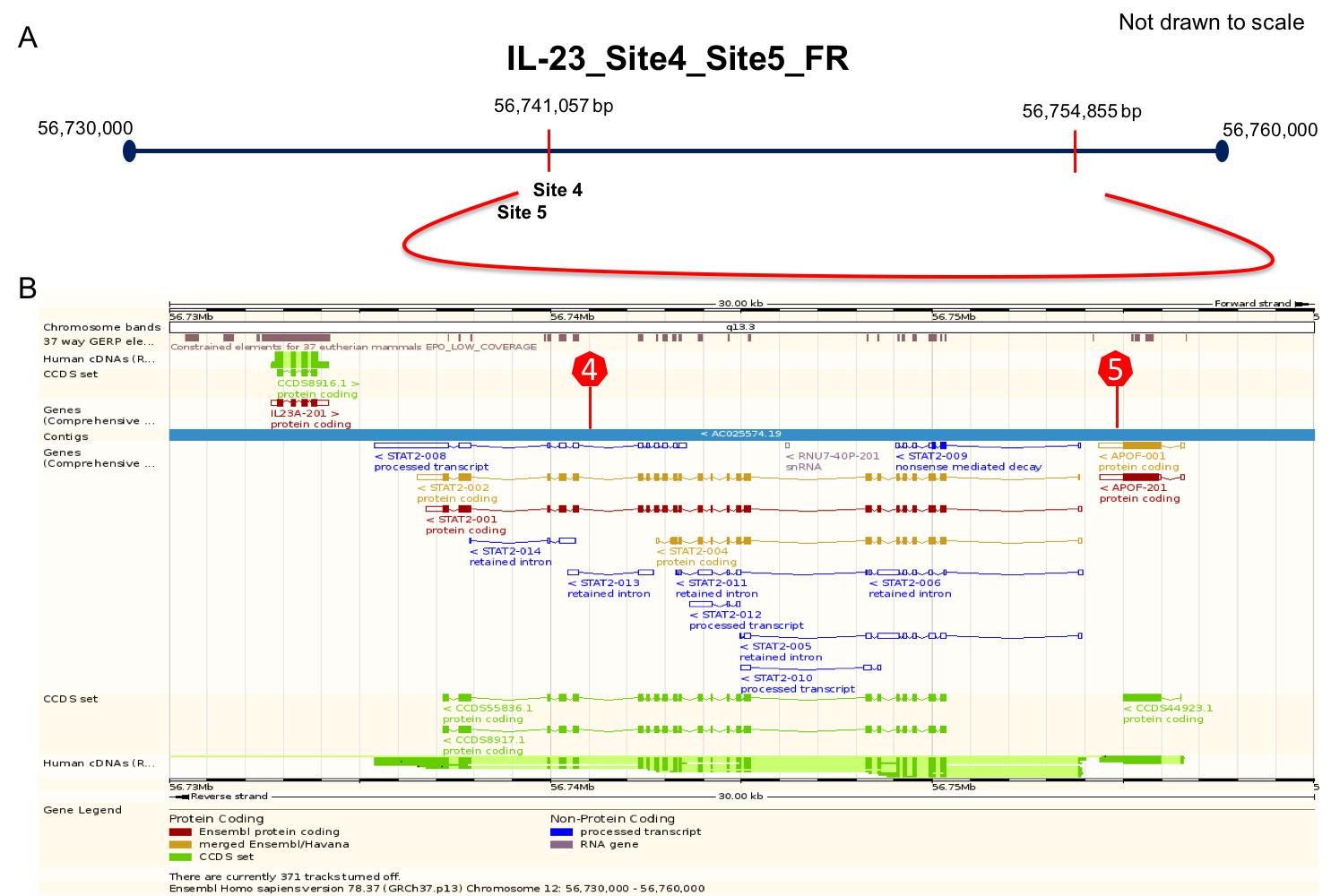


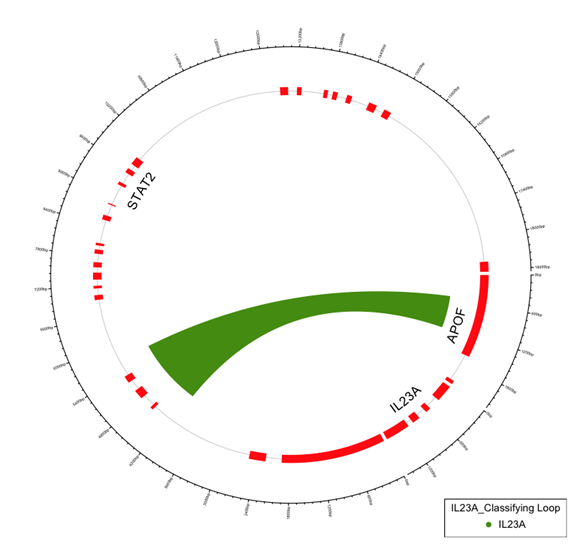
C

**Additional Figure S6. Graphical representation of the genomic co-ordinates of the IL-23** **CCS marker associated with non-response to MTX.** (A) The line represents the region on chromosome 12 from 56,730,000 to 56,760,000 with the two EpiSwitch co-ordinates (Red) that form the chromosomal loop. Orientation of EpiSwitch site/loop is indicated by FR (forward–reverse). (B) The Ensembl browser view of the IL-23 gene on chromosome 12 with the EpiSwitch^TM^ sites marked with red symbols. (C) Circos plot of the IL23 CCS marker showing the chromosomal loop (green).


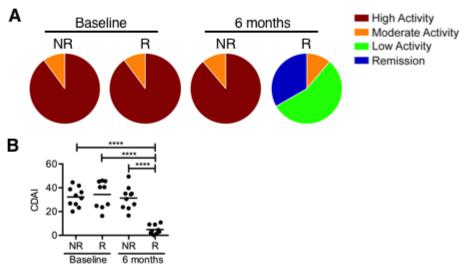


**Additional Figure S7**. **Clinical characteristics of independent blinded validation cohort.** The Blinded validation cohort of responder (R) and non-responder (NR) RA patients were selected based on DAS28 EULAR response criteria (see methods). (A) Pie charts show the clinical interpretation of CDAI scores for both R and NR patients at baseline and six months. (B) CDAI scores of R and NR patients at baseline and six months. *****P*<0·0001 by Kruskal–Wallis test with Dunn's multiple comparison post-test.

**Additional Table S9.** **CCS markers associated with eQTLS**

| **Marker** | **Chr** | **CCS Chr location** | **Class** |  | **Number of mapped eQTLs** | | |  |
| --- | --- | --- | --- | --- | --- | --- | --- | --- |
|  |  |  |  | **100bp** | **200bp** | **500bp** | **1000bp** | **200bp** |
| IFNAR1 | 21 | 34696683-34697716 | R | 3 | 6 | 8 | 9 | 16 |
|  |  | 34746261-34748821 |  |  |  |  |  |  |
| IL-21R | 16 | 27367632-27368855 | R | 9 | 11 | 11 | 16 | 20 |
|  |  | 27460578-27462829 |  |  |  |  |  |  |
| IL-23 | 12 | 56739503-56741059 | R | 2 | 3 | 4 | 4 | 7 |
|  |  | 56754853-56755609 |  |  |  |  |  |  |
| IL-17A | 6 | 52026495-52036963 | NR | 0 | 0 | 0 | 0 | 0 |
|  |  | 52049430-52051865 |  |  |  |  |  |  |
| CXCL13 | 4 | 78431566-78433247 | NR | 0 | 0 | 0 | 0 | 0 |
|  |  | 78523779-78526587 |  |  |  |  |  |  |

eQTL data was obtained from the publicly available data generated by Walsh et.al., 2016 (4)


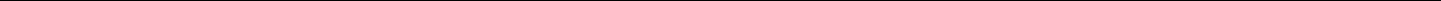


*CCS* Chromosomal conformation signature, *eQTL* expression of Quantitative Trait Loci, *Chr* chromosome, *GWAS* genome-wide association studies, *SNP* single nucleotide polymorphism.

**Additional References**

1. Rau R, Herborn G (2004) Benefit and risk of methotrexate treatment in rheumatoid arthritis. *Clin Exp Rheumatol* 22:S83–S94.

2. Ray P, Le Manach Y, Riou B, Houle TT (2010) Statistical evaluation of a biomarker. *Anesthesiology* 112(4):1023–1040.

3. Dekker J, et al. (2002) Capturing chromosome conformation. *Science* 295(5558):1306–11.

4. Walsh AM, et al. (2016) Integrative genomic deconvolution of rheumatoid arthritis GWAS loci into gene and cell type associations. *Genome Biol* 17(1):79.
